# The Unique Photophysical Properties of the Peridinin-Chlorophyll-a-Protein

**DOI:** 10.2174/1389203715666140327111139

**Published:** 2014-06

**Authors:** Donatella Carbonera, Marilena Di Valentin, Riccardo Spezia, Alberto Mezzetti

**Affiliations:** 1 Dipartimento di Scienze Chimiche, Università di Padova, Via Marzolo 1, 35131 Padova, Italy;; 2 CNRS, UMR 8587, Laboratoire Analyse et Modélisation pour la Biologie et l’Environnement, Université d’Evry-Val-d’Essonne, Bd F. Mitterrand, 91025 Evry Cedex, France;; 3 Laboratoire de Spectrochimie Infrarouge et Raman LASIR, UMR 8516 CNRS, Bat. C5, Université Lille 1, Cité Scientifique, 59655 Villeneuve d’Ascq, France;; 4 SB2SM, UMR 8221 CNRS Université Paris 11, IBITeC-S, Bat 532, CEA-Saclay, 91191 Gif-sur-Yvette cedex, France

**Keywords:** Carotenoids, light-harvesting, peridinin, peridinin-chlorophyll-protein, photoprotection.

## Abstract

Peridinin-Chlorophyll-a-Proteins (PCPs) are water-soluble light harvesting complexes from dinoflagellates. They have unique light-harvesting and energy transfer properties which have been studied in details in the last 15 years. This review aims to give an overview on all the main aspects of PCPs photophysics, with an emphasis on some aspects which have not been reviewed in details so far, such as vibrational spectroscopy studies, theoretical calculations, and magnetic resonance studies. A paragraph on the present development of PCPs towards technological applications is also included.

## 1. INTRODUCTION

Light-harvesting (LH) complexes are used by photosynthetic organisms to increase the overall efficiency of photosynthesis. Light is collected by LH complexes; subsequently, energy is funneled to the reaction center, where it is converted into electrochemical potential. Dinoflagellates, unicellular algae constituting one of the most important classes of phytoplankton, use a water-soluble LH complex called peridinin-chlorophyll-*a*-protein (PCP) with a 4:1 peridinin/chlorophyll ratio (note: PCPs with different stoichiometry also exist, as we will discuss later) [1]. The presence of peridinin (Per) molecules in the PCPs enables the organism to collect light in the visible spectral region where chlorophyll (Chl) poorly absorbs [2]. The absorbed light energy is then transferred to Chl *a* with high efficiency through energy transfer. Per has also an important photo-protective role as it can quickly and efficiently quench the Chl *a* triplet state, which could be a source of singlet oxygen.

After the pioneering works of Song and coworkers [3, 4] PCPs have been the subject of an increasing number of publications in the last two decades, using different techniques ranging from magnetic resonance to static and time-resolved (fast and ultrafast) electronic and vibrational spectroscopies. This strong interest from the biophysical community is not only related to the biological relevance of PCPs, but also to its peculiar photophysical behavior and to the availability of high-resolution X-ray structures [1, 5-8]. Furthermore, PCPs have found application in biomedical optics [9 and refs therein], and an increasing number of works reports its use in hybrid nanostructures for light harvesting (e.g. [10]). 

This review aims to provide a state-of-the-art description of the photophysics of PCP complexes and it is organized as follows. Following the introduction, section 2 and 3 describe the physicochemical and spectroscopic properties of PCP pigments (Per and Chl). In section 4 the different types of PCPs discovered so far will be presented. In section 5 the different steady-state spectroscopic properties of PCPs will be described and related to the difference in structure among PCPs. In section 6 we will briefly summarize the present knowledge of the energy transfer between Per and Chl; this topic has been the object of a recent review [2] and only the main points and newest literature will be presented. Section 7 will cover a topic for which no reviews are available in the literature, i.e. the photoprotection mechanisms in PCPs. In section 8 the technological applications of PCPs and of Per will be described. Finally, in section 9 we will present the main conclusions and perspectives and we will underline the most “hot” topics (notably the questions that still need to be answered to fully understand PCP photophysics).

In the present review we put a particular emphasis on vibrational and EPR spectroscopies and theoretical calculations since they complete the typical electronic spectroscopy studies that were recently reviewed [2]. We will show how these additional methods are extremely appropriate to investigate key issues of PCP photophysics, such as Per electronic structure, protein dynamics, triplet formation, and pigment-protein interactions.

## 2. PERIDININ

### 2.1. Structure 

**Fig. (1):**
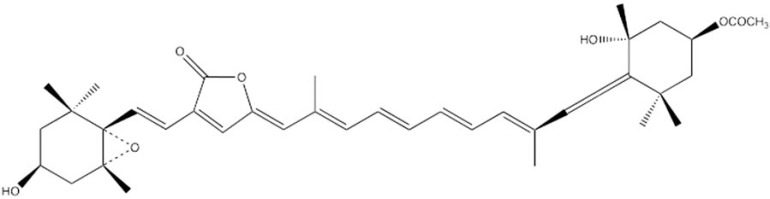
Structural formula of peridinin (Per).

The molecular structure of Per shows several peculiar features. Instead of the C40 system present in most carotenoids, Per has an unusual C37 skeleton. An allene moiety and a lactone ring are conjugated with the π-electron system of the carotenoid backbone. An ester group is located on one β-ring with a tertiary alcoholic group, and an epoxy group with a secondary alcohol on the opposite β-ring (see Fig. **1**).

### 2.2. Electron Spectroscopy

**Fig. (2):**
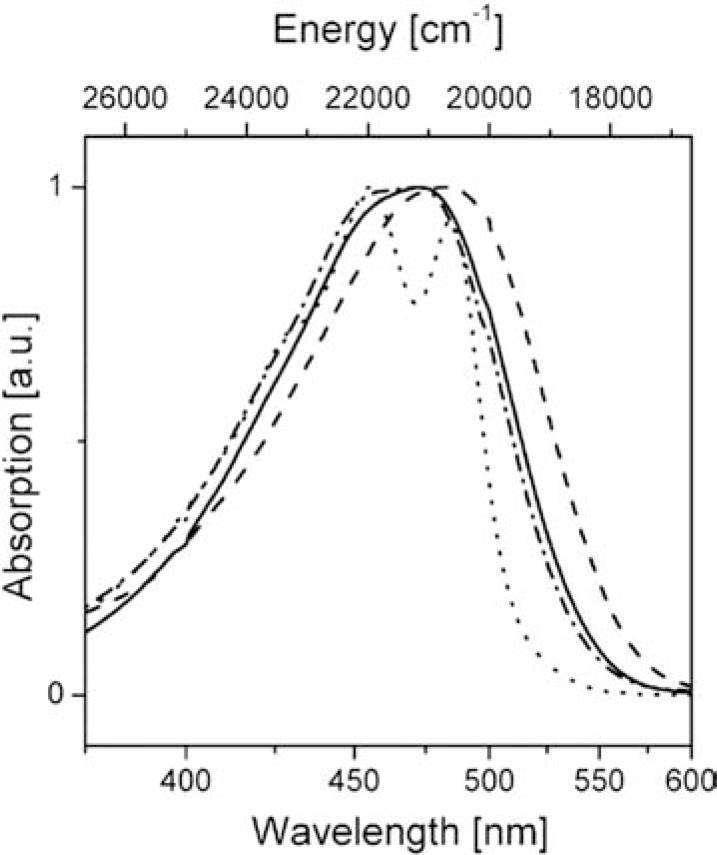
Absorption spectra of Per in n-hexane (•••), acetonitrile (− • −), methanol ( ), and ethylene glycol (− − −). All spectra are normalized. Reprinted with permission from Zigmantas, D. ; Hiller, R.G. ; Yarsev, A. ; Sundstrom, V. ; Polivka, T. J. Phys. Chem. B 2003, 107, 5339-5348 Copyright 2003 American Chemical Society.

In general, the photophysical properties of carotenoids are determined by their structure, essentially by the length of the π-electron system and the nature of the functional groups attached to the polyene chain [11]. Normally, the conjugated chain of carotenoids belongs to the C_2h_ point group. In this case the S_0_ (ground state), S_1_ and S_2_ states have symmetry ^1^A_g_^-^, ^2^A_g_^-^ symmetry and ^1^B_u_^+^, in the C_2h_ point group, resulting in a forbidden S_0_ to S_1_ transition [12]. Conversely, the S_0 _– S_2 _transition is symmetry-allowed and consequently there is strong absorption in the 400-550 nm region. As a consequence of the forbidden character of the S_0_ - S_1_ transition, fluorescence from the S1 state is normally extremely weak. Carotenoids with a carbonyl group in their conjugated chain, such as Per, show a large deviation from the ideal C_2h _symmetry and have peculiar photophysical properties, such as a relatively high quantum yield of S_1_ fluorescence [13]. In the case of Per, a fluorescence quantum yield of 10^-3 ^and a S_1 _lifetime is ~160 ps are observed in non-polar solvents. These values drop to 10^-5 ^(for the fluorescence quantum yield) and ~10 ps (for the S_1 _lifetime) in polar solvents [14]. The shortening of the Per lifetime in polar solvents is due to another excited state called Intramolecular Charge Transfer (S_ICT_) with a strong charge transfer character (see also section 2.5 and ref. [2])). In apolar solvents Per shows a typical 3-peak absorption band but in polar solvents this is replaced by a broader, structureless band due to the loss of vibronic structure (see Fig. **2**).

Time-resolved spectra in the femtosecond domain also show dramatic polarity-induced changes in the spectra. The transient absorption spectrum in n-hexane is characterized by a narrow excited state absorption at 510 nm, attributed to a S_1_-S_n_ transition; at higher wavelengths, in the NIR region, a signal between 1200 and 1800 nm, characteristic of the S_1_-S_2_ transition of carotenoids, is observed (see [2] and refs. therein).

Conversely, the transient absorption spectrum in methanol shows a strong and broad positive band centered at 590 nm, attributed to the S_ICT_-S_n_ transition, whereas in the NIR region, beside the S_1_ - S_2_ transition positive signal between 1200 and 1800 nm, a strong negative spectral band appears at ~950 nm. The 950 nm band is attributed to a stimulated emission from the S_ICT_ state (see [2] and refs. therein).

Further insight on the Per photophysics was obtained through the study of newly-synthetized compounds with a structure similar to Per, with modified key structural features (see [15, 16] and refs. therein). Several key points emerged: 

1) The peculiar structure of Per provides the strongest dipole moment of the excited state among all the investigated molecules. The presence and the position of the lactonic ring [17] of the allenic moiety [18] and of the C37 skeleton [19] are key features which maximize the charge transfer character of the excited state; 

2) The lactonic ring maintains the stereochemistry of the conjugated double bonds in an all-trans configuration [20] resulting in maximal absorption in the blue-green region of the visible spectrum; 

3) The S_ICT_ state is most probably highly localized in the lactonic ring [19];

4) It is suggested that the allene group acts as the electron donor in the charge transfer process following photoexcitation [18];

5) In Per, S_1_ and S_ICT_ most probably act as independent states [15, 21].

### 2.3. Vibrational Spectroscopy

The above-mentioned results show that the solvent (or, more broadly speaking, the surrounding environment) can strongly modify Per photophysics. To better characterize the molecular structure of Per in the fundamental and excited states in different solvents and to identify the intermolecular solute-solvent interactions through which this peculiar effect takes place, vibrational spectroscopy experiments on Per in various solvents have been performed [22-24]. The main conclusions are: 1) the stretching mode of the lactonic C=O moiety is sensitive to solvent polarity and to the H-bonding donation properties of the solvent, with values ranging from 1736 cm^-1^ (methanol) to 1777 cm^-1^ (cyclohexane) [22]; 2) solvent dynamics (notably through the formation/disruption of H-bonds, see later) strongly influences the C=O bandshape [22]; 3) the position of C=C stretching modes between 1520 and 1650 cm^-1^ and of the allene stretching (1927-1930 cm^-1^) are essentially solvent independent [22]; 4) the vibrational signatures of the S_1_ and S_ICT_ states appear to be rather similar [23]. 

### 2.4. Theoretical Studies on Per Ground State

Several studies have been performed to understand Per properties from a theoretical point of view. The Per structure was obtained following two main strategies: (i) using information of X-ray structures, available since the work of Hoffman[5] which opened the way to many theoretical calculations and (ii) performing full optimization from computer-built structures. It is important to notice that Per in PCPs is in all-trans conformation, while in solvents cis structures can be populated (in particular in non-polar solvents) [25]. Thus different authors have performed optimization of Per starting from X-ray structure, using different strategies to keep the structure as close as possible to the original in the protein, and have compared properties with those obtained from fully optimized geometry [25-27]. To this aim, semi-empirical Hamiltonian were initially employed [25], but now thanks to advances in computational power and code developing, Density Functional Theory (DFT) calculations are ordinarily performed [22, 26-28].

The structural characterization of Per obtained from theoretical calculation can be compared to experiments mainly through vibrational spectroscopy that directly probes the molecular structure and the effect of the environment. Recently “static” DFT calculations (i.e. calculations where the molecular geometry is minimized with respect to energy and then vibrational frequencies are obtained) have been supplemented with dynamical DFT-based simulations from which vibrational modes can also be extracted [22]. This was particularly important in order to study local solvation effects. While the implicit solvation methods, used in “static”-DFT, are able to provide vibrational frequency shifts due to differences in solvent polarity, only “dynamical”-DFT with an explicit solvent description was able to correctly describe the role of solvent-Per H-bond involving the lactonic carbonyl responsible for a further red shift of the C=O stretching band [22]. The investigation of vibrational properties was required to attribute peaks of lactonic carbonyl and polyene chain. The lactonic ring is a peculiar moiety of Per and can be used as probe for Per in a complex environment where other C=O groups are present, since its signal can be obtained separately, as done in Resonance Raman experiments, ultrafast IR experiments [23, 24] and triplet-minus-singlet step-scan FTIR experiments. [29-31]. As already mentioned, the C=O stretching of Per is highly sensitive to the environment, due to bond polarity and also to the possibility of acting as a H-bond acceptor [22]. Thus, through a coupled simulation/experimental study, it was suggested that the differences found as function of the solvent (apolar, polar/aprotic, polar/protic) can be used in assigning properties of Pers in PCP that have different environments [22]. The vibrational bands obtained for Per in solid state and in solution are reported in Table **1**.

### 2.5. Theoretical Studies on Per Photophysics

As mentioned before, Per belongs the family of carotenoids for which spectroscopic properties are mainly determined by the symmetry of the conjugated chain.

Excited-state calculations were performed following two strategies: Time Dependent TDF (TD-DFT) [26, 27] or semi-empirical methods, in particular MNDO-PSDCI [25] recently enriched by EOM-CCSD methods on reduced Per system (the full system is computationally untreatable at this level of theory at the present time) [32]. A much-debated question of excited states properties is the presence and the nature of a S_ICT_ state, suggested from the solvent-dependent photophysical properties of Per (see section 2.2 and refs. [33, 34]). The enhancement of S_ICT_ is obtained when Per is placed in protic solvents, like methanol or ethylene glycol, due to hydrogen bonding via the carbonyl group in the lactone ring that is part of the delocalized π system.

The S_ICT_ state was found to be stabilized in PCP and suggested to be a key property for highly efficient transfer of energy from Per to Chl [35-39].

Vertical, Franck-Condon, transitions of Per agree on the order and on the pseudo-symmetry of vertical states. In other words, S_1_ has an ^1^A_g_^-^ character and S_2_ an ^1^B_u_^+^ one (as previously remarked they are “symmetry-like” states). Thus for the nature of the S_ICT_ state different models were proposed: (i) the S_ICT_ state is distinct from S_1_ [14, 24, 37]; (ii) the S_ICT_ and S_1_ are coherently mixed (or the same state); [36, 40-42]; (iii) the S_ICT_ state is the S_1_ state with a large intrinsic dipole moment [25]; (iv) S_ICT_ is formed by mixing S_1_ and S_2_ state moving away from Franck-Condon portion of the S_1_ excited state surface [32].

At this point we need to clarify some aspects of electronic states. S_n_ states (S_1_, S_2_ etc. …) are adiabatic states obtained by Hamiltonian diagonalization, and thus to recover chemical concepts expressed in terms of “Valence Bond” picture, one has in principle to project the adiabatic states on the diabatic states defined in some way. These procedures have never been performed on Per, due to the complexity of the system, but qualitative characterizations of excited states were performed from the analysis of orbitals involved in the transitions and excited states dipoles. Some differences in characterization of S_ICT_ arise from the misleading use of adiabatic (S_1_, S_2_, etc …) and diabatic states, and then the 4-model scenario described previously can be simplified in two models: (i) S_ICT_ is present at Franck-Condon region and is stabilized by solvent polarity; (ii) S_ICT_ is obtained evolving on S_1_ and by the mixing of diabatic states with ^1^A_g_^-^ and ^1^B_u_^+^ character. Excited states dynamics of Per and related systems suggest this last picture [16]. Excited state energies are summarized in Table **2**. 

From theoretical side, the first excited state calculations were reported in 2003 by Shima *et al.* [25] using semi-empirical methods and by Vaswani *et al.* using TD-DFT with Tamm-Dancoff approximation [26] followed by Spezia *et al.* [27] in 2004 where the excited states calculations were coupled with Per dynamics (at ground states). TD-DFT methods agree that the molecular motion can be responsible of the inversion between states. From a methodological point of view TD-DFT is known to reverse the A_g_^-^ B_u_^+^ order [43] and some agreement is obtained by employing Tamm-Dancoff approximation even if this can be considered fortuitous [26]. Semi-empirical, MNDO-PSDCI methods are in good agreement with experiments [25, 32, 44, 45] as well as EOM-CCSD methods [17, 32] even if the doubly excited dark state, A_g_^-^, can be poorly described by these last methods as suggested by Krylov [46]. 

The final picture can be thus summarized as follows: after UV-Vis absorption the system is excited to S_2_ that quickly interconverts into S_1_ (here we can assume that the geometry is not dramatically changed), or it can transfer the energy to the Chl (this is the case of Per in PCPs; we will discuss energy transfer in section 6). Then, through excited states dynamics the S_1_ that has ^1^A_g_^-^ symmetry in Franck-Condon region evolves towards a region on the excited state surface where the electronic density changes into an S_ICT_ state. This is obtained through a mixing between states and the dynamics are tuned by the polarity of the environment that modifies the barrier between the Franck-Condon geometry and the S_ICT_. This state can either relax to the ground state or play its photochemical role, by transferring its energy to Chl (in PCP, see section 6). 

## 3. CHLOROPHYLLS

### 3.1. Electronic Properties of Chlorophylls: Singlet State

In this section the electronic structure of Chls and bacteriochlorophylls (BChls) and how a solvent/protein environment can finely tune their excitation characteristics, will be briefly described. These molecules are characterized by a Mg^2+^ ion coordinated by a chlorin or bacteriochlorin ring, containing four pyrrole-like units with some differences. In chlorin, only one of the pyrrole is reduced, while in bacteriochlorin there are two reduced rings. An exception is Chl *c*, which does not contain reduced rings. A common feature of all natural Chl and BChl pigments is an attached cyclopentenone ring, which is called the isocyclic ring. Also, the so-called phytyl chain, a polyisoprenoid alcohol chain esterified to the pyrrole ring D, is a common structural element, although in some BChls this group is esterified to other alcohols. Further structural differences concern the other substituents at the rings. 

Chl *a* and Chl *b*, are the chlorophylls found in higher plants. In Chl *d*, a chlorophyll form found in cyanobacteria, the divinyl group in ring A of Chl *a* is replaced by a formyl group. An overview of natural chlorophyll pigments is given in Fig. (**3**).

The electronic structure of (B)Chls is described in a qualitative way by the Gouterman Four-Orbital Model. This model, based on configuration interaction, qualitatively explains the absorption spectra of (B)Chls and defines the nomenclature of the absorption band observed [47, 48].

In porphyrins, due to the D_4h_ molecular symmetry, the lowest unoccupied molecular orbital (LUMO, e_g_) is twofold degenerate. The two highest occupied molecular orbitals (HOMO-1 and HOMO, a_1u_ and a_2u_) are near degenerate. The configurations arising from excitation of an electron from the HOMO or HOMO-1 to the LUMO are near degenerate and interact strongly. This configuration interaction gives rise to two states and therefore to two absorption bands, the weak Q band and the intense B (or Soret) band. In lower-symmetry porphyrin derivatives like Chls, the degeneracy is removed and the Q and B bands are split into two transitions, which are polarized within the plane of the macrocycle and are called Q_x_/Q_y_ and B_x_/B_y_. The Q_x_ band is usually much weaker than the Q_y_ band. For Chl *a* and *b* it was proposed that the B_x_ and B_y_ bands are accidentally degenerate and are thus not resolved in most experimental spectra. The difference in intensity between the Q and B bands is much smaller for chlorophyll derivatives than for porphyrins. More recently, several theoretical methods have been applied for the description of the absorption spectra. These methods include semiempirical and *ab initio* CI methods and linear response time-dependent SCF methods (TDHF and TDDFT) [49, 50].

**Fig. (3):**
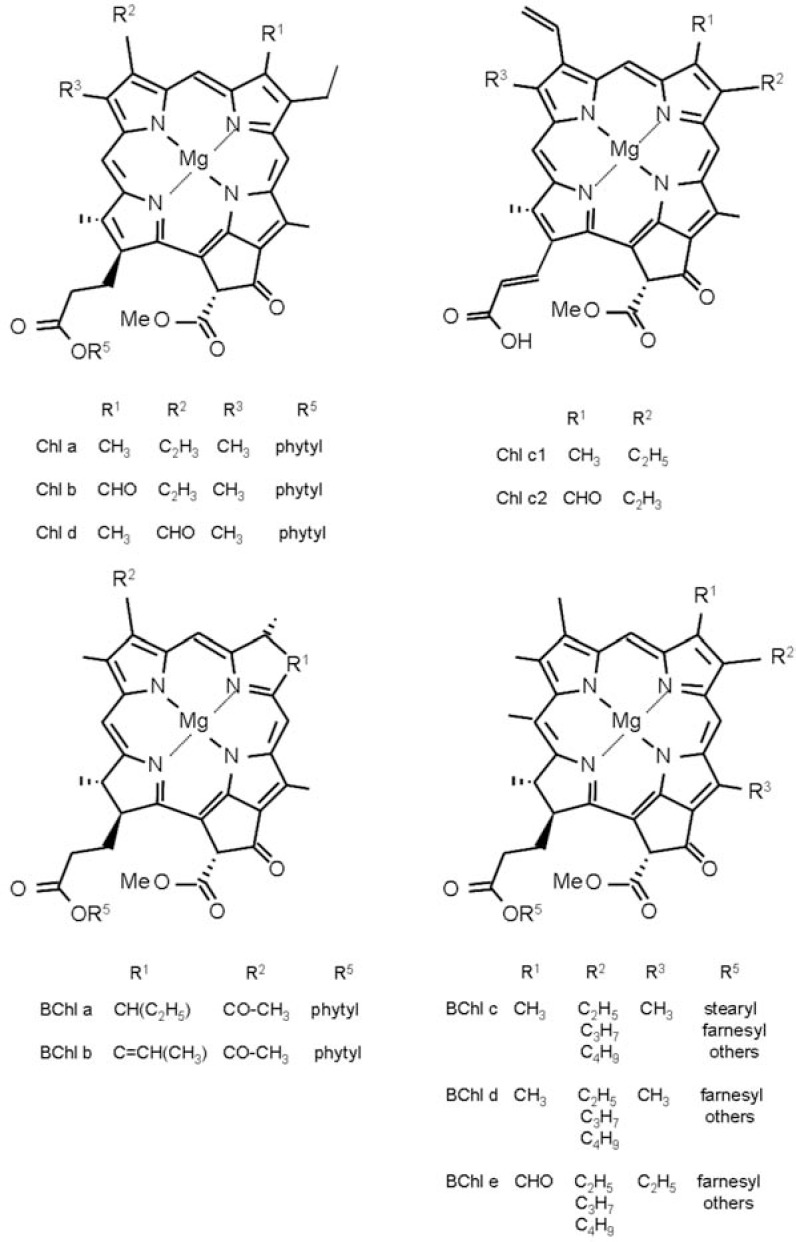
Molecular structures of natural Chl pigments.

A comparison of the absorption spectra of Chl* a* and *b*, the commonest form of Chls found in photosynthetic plants, shows that the Q band is increased and the B band is lowered in energy due to the carbonyl group present in Chl *b* (see Fig. **3**). This trend is correctly predicted by some theoretical methods, namely ZINDO/S CIS, PM5, and CIS, but is not clearly reproduced with TD-DFT (SAOP/TZP, B3LYP/ TZVP, and B2PLYP/TZVP) and CIS(D)/TZVP [51].

Chl *d* differs markedly from all the other chlorophylls in having its Q_y_ transition in the near infrared (710 nm), rather than in the red. Therefore, from this point of view, it is similar to BChls, which also have Q_y_ transitions in the infrared region. Chl *c *absorbs, in organic solvent, in the blue with a Q_y_ peaks at 633 nm.

The absorption spectrum of all (B)Chls is characterized by a well-resolved vibrational structure. The absorption spectra of (B)Chls are very sensitive to solvent and protein effects. Both the dielectric constant of the solvent and its coordinating properties to the central Mg affect the transition energies [52-55] In Chls the central Mg^2+^ usually has a five or six coordination [56]. In protic solvent H-bonding to carbonyl groups may also have a strong effect on the electronic transitions [57, 58]. The oscillator strengths of chlorophyll pigments are also dependent on the solvent. For instance, Chl *a* shows a range of the extinction coefficient going from 6.14x10^-4^ mol^-1^cm^-1^, in cyclohexane, to 8.52 x10^-4^ mol^-1^cm^-1 ^in triethylamine.

In protein complexes, the (B)Chls are bound in anisotropic environment, which determines geometric and electronic properties. Long-range electrostatic interactions affect also the site energies of the pigments [53].

### 3.2. Chlorophyll Triplet State 

Following excitation to one of their singlet states, (B)Chls return to their electronic ground states (S_0_) through internal conversion, radiative decay (fluorescence emission), vibrational relaxation and inter-system crossing (ISC). This last process involves ISC to the lowest excited triplet state (T_0_) and from T_0_ to the S_0_.

The triplet state carries two unpaired electron spins resulting in a net electron-spin angular momentum S=1. Three triplet spin sublevels are connected with this state and because of dipole-dipole interactions they are non-degenerate, even in zero magnetic field [59, 60 and refs. therein]. The lifetime of T_0 _is long (about 1 ms) with the three spin sublevels (x, y, z, referring to the zero-field axis system) having different kinetic constants. Chls have broad triplet-triplet absorption bands in the range 475-600 nm [61]. Except by its kinetics, T_0_ can also be characterized making use of the fact that this carries two unpaired electrons, resulting in a net electron-spin angular momentum S=1. Because of the dipole-dipole interaction between the two electron spins, the three sublevels are not degenerate and the energy difference among them can be described by the ZFS (zero field splitting) parameters D and E. Theoretically the ZFS parameters are related to the spatial distribution of the unpaired electrons and depend on the solvent and coordination at the Mg. For an extensive review, see [62]. The sublevel corresponding to the z direction of the dipolar interaction, considered the perpendicular to the porphyrin plane, shows the slowest decay and populating rates, while the decay and populating rates of the in-plane x, y sublevels are similar. The sublevel kinetics of (B)Chl triplet states are quite sensitive to the presence of the central metal and its ligation state [60]. The introduction of heavy metal such as Zn into the Chl ring strongly increases the ISC to the z level [60].

Chl triplet state formation have been studied also by FTIR spectroscopy (see [30, 63, and refs therein] and Resonance Raman spectroscopy (see [64] and refs therein) leading to the identification of vibrational marker bands for Chl and ^T^Chl states. These will be discussed in section 7, where the use of vibrational spectroscopy in the study of triplet formation in PCPs will be described.

## 4. DIFFERENT PCPs STUDIED

### 4.1. Natural PCPs

**Fig. (4):**
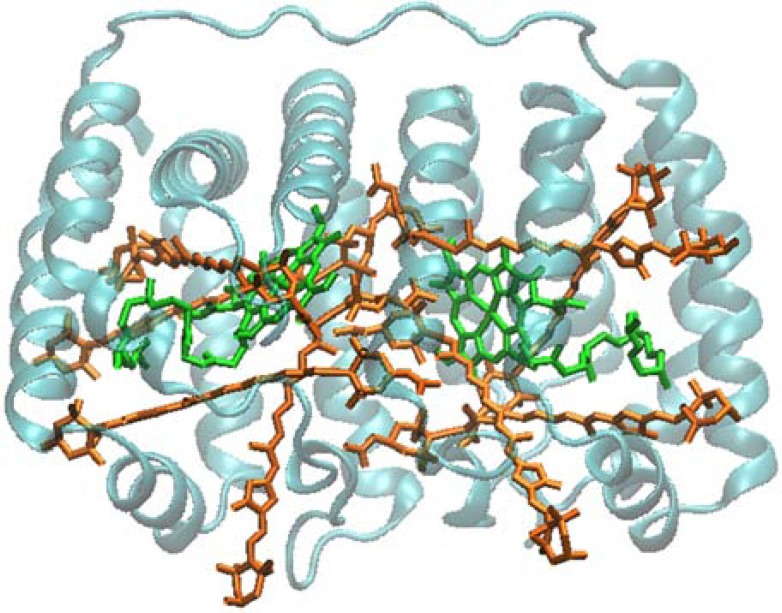
Structure of the MFPCP. Pers are shown in orange, Chl a in green. (PDB entry 1PPR).

Several different PCPs have been studied spectroscopically. The most studied is the so-called main-form from *Amphidinium carterae* (often called MFPCP), whose crystal structure has been solved in 1996 [5] to 2.0 Å, and recently to 1.35 Å [1]. The 2.0 Å structure was also the first PCP structure to be solved, and prompted a large series of spectroscopic studies. The structure showed a trimer of protein subunits with densely packed pigments. The pigments in each subunit are arranged as two pseudo-identical domains of four Pers and a Chl *a* molecule. Within a single domain range, distances between Pers are in the 4-11 Å range, and conjugated chains of Pers are in van der Waals contact with the tetrapyrrole ring of the Chl *a*. Distance between the Mg atoms of the two Chl *a* of the subunit is 17.4 Å. The pigment arrangement in MFPCP is shown in (Fig. **4**).

Beside the main form of the PCP complex described above, a second form, called High-salt PCP (HSPCP) was identified and crystallized. HSPCP has an apoprotein of different molecular mass (34 kDa vs. 32 kDa of the MFPCP) and only 31% identity in amino acid sequence. The most striking difference however is in the pigment composition, as HSPCP contains only 6 Pers and 2 Chl *a* molecules. The HSPCP structure at 2.1 Å was published in 2009 [7], and it showed a similar overall topology to MFPCP. The pigment arrangement is also somewhat similar, but two Pers (the 612/622 pair) of MFPCP are missing. For this reason, the comparison of spectroscopic results from MFPCP and HSPCP is particularly interesting. 

HPPCP (the PCP form from *Heterocapsa pygmaea*) has an apoprotein of 15.5 kDa molecular mass. The structure has not yet been published, but it has already been reported that HPPCP forms a stable homo/heterodimer of identical topology to MFPCP [1]. It has been characterized by several optical spectroscopy techniques [65]. There are also reports of PCP forms from *Symbiodinium* (where a Per to Chl *a* ratio of 4 is observed; a detailed spectroscopic investigation has been also reported [66]), and from *Alexandrium cohorticula*, which are reported to contains 10 to 12 Pers per 2 Chl *a* [67] although this is not consistent with the published absorption spectra [68]. MicroRaman *in vivo* studies have been reported for PCPs from *Gonyaulax polyedra* (now *Lingulodinium polyedrum*) [69] and *Pyrocystis lunula* [70]. 

### 4.2. Refolded PCPs

Recently, artificial PCP complexes have been produced and investigated in detail. This has been possible through the development of an *in vitro* reconstitution system of MFPCP [71]. Reconstitution systems for full-length MFPCP and for N-domain MFPCP exist. The latter (hereafter called RFPCP) presents the advantage that dimers of two identical domains are formed, reducing the heterogeneity of the pigment binding sites. Its structure has been solved, and it was found that the stable RFPCP homodimer is virtually indistinguishable from the MFPCP on the structural level. 

In addition, RFPCP with different Chls have been produced and their structure solved. Identical binding site were found for Chl *a*, Chl *b*, Chl *d*, with a slight displacement for BChl *a* [71].

### 4.3. Theoretical Studies on PCP

The resolution of MFPCP crystal structure in 1996 facilitated theoretical structural and dynamical studies of the pigment-pigment and pigment-protein interactions and their involvement in photophysical properties. Spezia *et al.*[72] studied the monomer of PCP with 4 Pers and 1 Chl by coupling molecular dynamics simulations with electronic properties of the Chl. Their calculations showed that the dynamical environment provided by the proteins and the Per is able to spread the absorption wavelength of Chl *a* of several wavelengths (14 nm difference for the average absorption with a maximum spread of 40 nm), suggesting a conformational regulation of the photophysical activity. In addition, the protein seems to have a bigger role than Pers in this wavelength spread. Interestingly, molecular dynamics show that one Per, Per614 - the one that is packed between Chl *a* and the protein structure - seems to be more rigid than the other three. From an energetic point of view Mao *et al.* [73] showed the importance of π-π stacking interactions between Pers and Chl and surrounding aromatic groups of protein residues that are responsible for the Pers bindings in PCP and which form the molecular basis of structural stabilization of carotenoids to form the pigment-protein complexes.

## 5. SPECTROSCOPIC CHARACTERIZATION OF PCP COMPLEXES

### 5.1. MFPCP

UV-Vis absorption spectra of MFPCP (both at room and low temperature) are dominated by the S_0_-S_2_ transition of Per, which give a broad absorption band between 400 and 550 nm; the narrow peak at 435 nm is due to the Soret band of Chl *a*. The absorbance of Per in PCP is without vibrational structure and resembles that of Per in polar solvents. Analysis of optical spectra at low temperature suggested that the Pers inside MFPCP have different spectral properties; spectrally distinct Pers with 0-0 transitions at 520, 537, and 555 nm were identified [74]. Similar results (with slightly different values: 518-523, 528-534, 543-545 nm) were obtained by alternative strategies [42, 75, 76]). For these three Pers, the spectra were identified as identical in both structural domains of the MFPCP. Conversely, the fourth Per appeared to have different spectral properties in the two domains, peaking as 485 and 465 nm respectively. These two Pers were assigned to Per 612 and Per 622 and designated blue-shifted Pers. 

### 5.2. HSPCP

The absorption spectrum of HSPCP shows some differences compared to the one of MFPCP. As expected, lower absorption is observed in the 400-550 nm Per region; at low T a spectrum with better resolved Per bands is also observed. As mentioned before, the two missing Pers in HSPCP – when compared to MFPCP - are Per 612 and 622, the so-called blue-shifted Pers of MFPCP. Nevertheless, a Per with blue-shifted absorption peak is still present, showing that the presence of at least one blue-shifted Per is a common feature of MFPCP and HSPCP complexes from *A. carterae. *A further interesting spectral feature of HSPCP is that for the Q_y_ band of Chl *a *a splitting observed at low temperature. It is interesting to note that both these spectral features – blue-shift of one Per and splitting of the Chl *a* Q_y_ band have been explained as consequence of the effect of a charge on specific amino acids [2]. 

### 5.3. Reconstituted PCP Complexes

PCP complexes reconstituted with Chl *b*, acetyl Chl *a*, Chl *d*, and BChl *a *(instead of Chl *a*) show systematically different spectral position of the Q_y_ spectral bands, making these complexes ideal for studying the mechanism of energy transfer between Pers and Chls [71]. 

## 6. ENERGY TRANSFER FROM PER TO CHL

### 6.1. Electronic Spectroscopy Studies 

The detailed understanding of energy transfer mechanisms in MFPCP is not yet fully understood. A detailed description can be found in the review by Polivka *et al.* [2] and in recent literature. Here we will just summarize the current knowledge of the subject and describe the contributions provided by ultrafast IR data. In PCP complexes the light is absorbed by Per and then transferred to the Chl with high efficiency [2]. When Per absorbs light, it is excited into the S_2_ state. Krueuger *et al.* [77] suggested that a certain percentage (25-50%) of energy is directly transferred from the Per S_2_ state to the Q_X_ state of Chl *a*. The rest of the energy transfer proceeds via the S_1_/S_ICT_ route. On the other hand, transient absorption studies by Frank and co-workers [40] and Akimoto *et al.*[78] concluded that the energy transfer occurs mainly via the S_1_ state of Per to Chl *a* Q_y_ level. This route was supported by an energy transfer model based on the X-ray structure of MFPCP that assumed a full Coulomb coupling between Pers and Chl [79]. Among the different Pers present in the MFPCP from *A. carterae* Per 612 seemed to have a particular role, i.e. to transfer the energy from its S_2_ state to S_2_ states of the other Pers. A recent study on RFPCP also clearly demonstrates the peculiar behaviour of another Per, Per 614 (the closest to the central Chl *a*) which has the strongest interaction with the central Chl* a* [6]. Finally, the excited state S_1_ dynamics leading to the S_ICT_ state may also be responsible for the highly efficient energy transfer, since the formation of a large dipole enhances the electronic coupling with Chl *a *[38]. 

### 6.2. Vibrational Spectroscopy Studies

A key point for a detailed investigation of PCP photophysics is the possibility to follow the behaviour in time of the different Pers through marker bands. Electronic transitions of Pers in PCP give strong overlapping bands. In contrast, vibrational bands of Per are very narrow and – for the lactonic C=O stretching - the position should depend strongly on the local protein environment (see section 2). Ultrafast IR spectroscopy represents therefore a unique technique to study the energy transfer in PCP as in can in principle disentangle the different contributions from the various Pers, by examining lactonic C=O vibrations. Bonetti *et al.* [24] suggested that large part of the energy transfer proceeds via S_ICT_ state, which is localized on Per 621 and Per 611 (and/or Per 623/613), and they assigned a lactone C=O stretching at 1745 cm^-1^ for the ground state of these Pers. For per 614/624 they nominated a band at 1720 cm^-1^ (and possibly at 1750 cm^-1^).

These values, however, do not match with the assignments of C=O stretching proposed by Bovi *et al.* [22] on the basis of the X-ray structure and of the comparison with vibrational spectroscopy studies of Per in solution coupled with QM/MM calculations. More precise C=O stretching calculations for Pers in PCP based on QM/MM studies of the whole protein are in progress (Bovi *et al.*, in preparation) and can in principle help to clarify this discrepancy.

## 7. ENERGY TRANSFER & PHOTOPROTECTION

### 7.1. Triplet-Triplet Energy Transfer

In addition to the light-harvesting function, Pers fulfil the important function of protecting the system against photo-oxidative damage by quenching ^T^Chl, formed under excess light conditions [80, 81]. ^T^Chl sensitises the formation of singlet oxygen, a powerful oxidizing agent that is capable of damaging the whole photosynthetic apparatus:

^T^Chl a^*^ + O_2_ → Chl *a* + ^1^O_2_^*^ (singlet oxygen formation)

By virtue of their low-lying triplet state, Per and more in general carotenoids having more than seven double bonds in the conjugated polyene chain, are able to quench ^T^Chl as well as singlet oxygen directly if it is formed:

^T^Chl a^*^ + Car → Chl *a* + ^T^Car^*^ (Chl triplet quenching)

^1^O_2_^*^ + Car → O_2_ + ^T^Car (singlet oxygen scavenging)

This photoprotective function is therefore essential for photosynthesis in an oxygen-containing environment.

In PCP, the triplet state of Chl *a* is formed via ISC from the corresponding singlet state. The Per triplet state (^T^Per) is populated by triplet-triplet energy transfer (TTET) from ^T^Chl *a*. Photoprotection by TTET occurs with 100% efficiency [40]. 

The TTET process, based on the Dexter exchange mechanism [82], can be formalised as a simultaneous double electron transfer between the lowest unoccupied molecular orbitals (LUMOs) and the highest occupied molecular orbitals (HOMOs) [83]. In the weak-coupling limit, the TTET process is a non-adiabatic process with a rate constant defined by the golden rule [84]:

k=2πℏ|VTT|2FCWDS

where FCWDS stands for the Frank-Condon weighted density of states and V_TT_ is the electronic coupling between the donor (D) and acceptor (A) triplet states, defined as:

VTT=〈ψLUMOD(1)ψHOMOA(2)|e2r12|ψLUMOA(1)ψHOMOD(2)〉

The TTET in PCP has been extensively studied by means of advanced optical spectroscopies and by Optically Detected Magnetic Resonance (ODMR) and Electron Paramagnetic Resonance (EPR). 

### 7.2. Optical Spectroscopy and ODMR Results

The formation of ^T^Per in PCP was first demonstrated by ODMR, by monitoring either the Chl *a* fluorescence [85], or the triplet-triplet absorption of Per [65]. No Chl *a* triplet states were detected in PCP by ODMR showing that Chl triplet state are transferred to Per with 100% efficiency. ODMR spectroscopy, correlating the magnetic properties of the triplet state with the optical properties of the system, assigned the zero field splitting (ZFS) parameters (D and E) of Per in a very precise way: two slightly different triplet components were determined showing |D|=0.0448cm^−1^, |E|=0.0044cm^−1^ and |D|=0.04586cm^−1^, |E|=0.0045cm^−1^. These parameters depend on delocalization of the unpaired electrons and reflect the distance and symmetry of the two singly occupied orbitals [86-88]. Due to the multiple transitions present in the spectra of MFPCP from *A. cartarae *and to the temperature dependence of the signals, it was suggested that intra-cluster triplet migration among Per molecules was taking place in the complex. However, further experiments, mainly by EPR spectroscopy, have shown that the triplet is localized on only one Per. Moreover, low temperature splitting of carotenoid triplet state, as detected by ODMR, and a temperature dependence of the ODMR signals similar to that observed in PCP, were detected also in other light-harvesting complexes and assigned to different local protein-carotenoid interactions [89].

Transient optical spectroscopy has been applied to PCP and the triplet states have been populated after laser flash excitation. The Triplet minus Singlet (T-S) spectra at different temperature have been detected [40, 74]. The dynamics of the Per triplet state in the PCP complex was determined to be 17 ± 7 ns for the rise and 10 ± 1 μs for the decay at room temperature [44]. The several bands present in the T-S spectra were assigned to four different Pers carrying the triplet state in PCP. This point remains however controversial.

In PCP, as observed in many other light harvesting complexes, the T-S spectrum is characterized by a bleaching around 668 nm reflecting Chl *a* absorption change due to the formation of a triplet state carried by a neighboring Per. The bleaching is present either in the room temperature T-S spectrum detected by transient optical spectroscopy [74], or in the T-S spectrum detected at 1.8 K by ODMR [65]. This interaction signal shows concerted dynamics with the carotenoid triplet [74] and has been discussed in terms of a Stark effect, but its exact nature remains still unclear. 

More recently time-resolved step-scan FTIR difference spectroscopy has been applied to the study of ^T^Per. Time-resolved step-scan FTIR difference spectroscopy is an ideal technique to investigate triplet formation in photosynthetic proteins [90, 91]. The technique has been used to study triplet formation in MFPCP from *A. carterae *([29, 31]; see also [92]) and HPPCP ([30]; see also [92]). The spectral analysis of the 298 K FTIR spectra obtained from MFPCP, lead to the conclusions that two components – with different decay kinetics - are present in the spectra, and that two different Per triplet states are produced [29]. The fast component (τ = 13 μs) originates from a triplet localised on a Per characterized by a lactonic C=O at 1745 cm^-1^ (in the ground state). The slow component involves one or two Pers (characterized by lactonic C=O at 1720 cm^-1^ and 1741 cm^-1^) depending on the excitation wavelength [29]. Beside signals arising from ^T^Per formation, also putative bands reflecting ^T^Chl *a *formation were identified [29]; interestingly, all the above-mentioned bands disappear with typical carotenoid triplet decay time constants [29]. This would imply that in PCP, while the triplet is mainly localized on the Pers, there is a significant involvement of Chl *a*. Moreover the fact that the time-resolved FTIR difference spectra depends on the excitation wavelength of the triggering laser flash seems to indicate that ^T^Per formation can proceed via different pathways [29, 92]. 

Step-scan FTIR spectra recorded al 100 K, while confirming the overall shape of the difference spectra, seem to indicate that at low temperature just one Per conformer (with a lactonic C=O at 1746 cm^-1^) is involved in triplet formation [31]. Mezzetti and Spezia suggested also a way to rationalise the presence at 298 K of different Per conformers, which would differ simply in the H-bonding state of the lactonic C=O [31]. 

Step-scan FTIR spectra of HPPCP at 298 K differed from those obtained from MFPCP, in showing the presence of a single kinetic component (τ = 10 μs), with an associated difference spectrum very similar to the fast component in MFPCP [30]. The authors concluded that there is significant involvement of Chl *a *in the triplet state in HPPCP as well as in MFPCP [30].

The results above are not in agreement with data obtained by advanced EPR techniques (see section 7.3 and section 7.4), and these discrepancies will be discussed in section 7.5.

### 7.3. Time-Resolved EPR and Spin Angular Momentum Conservation

Time-resolved EPR (TR-EPR) and pulsed EPR techniques, electron-spin echo techniques and pulse electron-nuclear double resonance (ENDOR), coupled with photo-excitation are the most appropriate tools to investigate the electronic structure of the pigments in the triplet state. These techniques can probe the magnetic properties of the triplet state. [93, 94].

From the triplet state spectra it is possible to extract information on the ZFS tensor and on the electron spin polarization. Electron spin polarization derives from selective population and depopulation of the triplet spin sublevels and for this reason is a fingerprint of the mechanism of formation of the triplet state. 

The Chl spin polarization is due to anisotropic population of the triplet state sublevels by ISC and the EPR spectrum consists of emission and absorption lines, whose position is determined by the ZFS parameters.

**Fig. (5):**
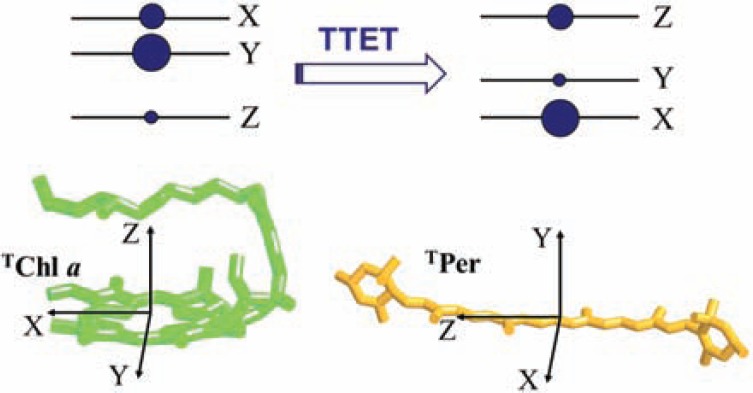
Schematic representation of electron spin polarization transfer in zero-field between the Chl a donor and the Per acceptor occurring during TTET. The size of the circles expresses the intersystem crossing population probabilities of the triplet sublevels for Chl a. The orientation of the ZFS tensor axes for Chl a and Per is reported. As an example, an hypothetical relative orientation of the ZFS is also represented, where the X,Y and Z axes of TChl a are respectively parallel to Z, X and Y axes of the ^T^Per.

The initial electron spin polarization of the Per triplet state is inherited from the Chl donor, due to spin angular momentum conservation during TTET [95-97]. Spin conservation is an intrinsic property of the electron exchange mechanism, which does not include spin dependent terms. The population probabilities of the donor triplet sublevels are transferred into the acceptor sublevels with relative probabilities given by the squared cosines of the angles relating the principal magnetic axes of the donor and the acceptor (see Fig. **5**). 

For this reason, electron spin polarization inherited by the acceptor triplet state depends exclusively on the relative geometrical arrangement of the donor–acceptor couple and on the relative population of the donor triplet-state sublevels.

**Fig. (6):**
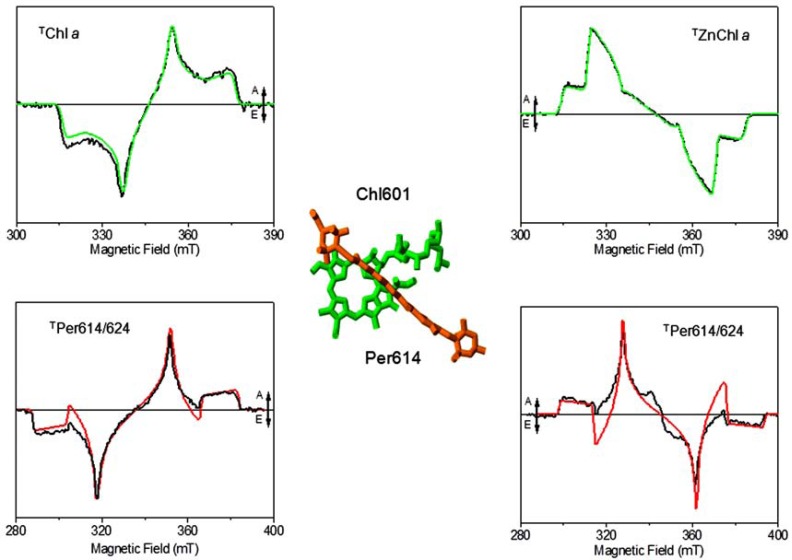
Left column from top to bottom: Powder TR-EPR spectrum of Chl a in 2Me-THF glass with relative simulation. Comparison between the calculated EPR spectrum of Per614/624 in MFPCP with the experimental powder TR-EPR spectrum of MFPCP. Right column from top to bottom: powder TR-EPR spectrum of Zn-Chl a in 2Me-THF glass with relative simulation. Comparison between the calculated EPR spectrum of Per614/624 with the experimental powder TR-EPR spectrum of ZnChl a-RFPCP. The geometrical arrangement of Chl a/Per614 pair the crystal structure of each couple is also reported. A = absorption, E = emission. The figure is based on the data from [97, 98], experimental conditions and simulation parameters reported therein.

In the first time-resolved EPR study on the ^T^Per by Di Valentin *et al.*, the concept of spin angular momentum conservation during TTET was exploited in order to investigate the mechanism of photoprotection in the MFPCP and HSPCP from *A.* *carterae* (see Fig. **6**) [98]. A specific path for TTET, common to both PCP forms, was demonstrated involving a specific Chl-Per pair (Chl601/602 and Per614/624). The information was derived by spectral simulation of the TR-EPR spin-polarized spectrum of the Per triplet state in the spin conservation framework. The simulation started from the sublevel population probabilities for Chl *a*, reported in studies of ^T^Chl *a in vitro *[99] considering the directions of the ZFS axes with respect to the molecular frames of the two partners of the TTET [100, 101] and the relative positions among Chls and Pers in the X-ray structure. 

**Fig. (7):**
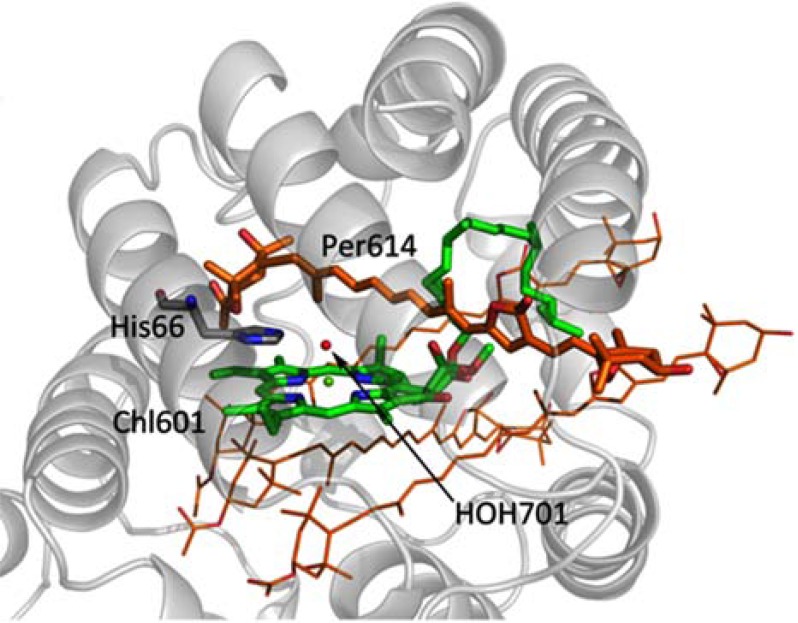
Highlight of the molecules of the photoprotective site in the NH2-terminal domain of PCP complex from A. carterae: Per 614, Chl a 601, the water molecule HOH701 coordinated to Chl a 601 and hydrogen-bonded to the histidine residue His66 (PDB entry 1PPR).

Since the polarization pattern of the TR-EPR spectrum remains unchanged up to physiological temperatures, the relevant structural requirements for efficient TTET were provided by the spectroscopic data. The EPR results pointed out that only one of the four Pers surrounding each Chl *a* of the pigment cluster is devoted to photo-protection. The identified Per molecule is distinguished by a smaller centre-to-centre distance to chlorophyll compared to that of the other Per molecules of the cluster (it should however be kept in mind that all four Per molecules are at van der Waals distance from the chlorophyll ring). The unique feature of the Chl601-Per614 (and equivalently Chl602-Per624) pair is the presence of the water molecule (HOH701 and HOH678) interposed between the two pigments acting as the fifth ligand of the chlorophyll’s Mg ion, while no bridging molecules are present at the interface between the Chl and the other Pers (see Fig. **7**). Two highly conserved histidine residues (His66 and His229) are hydrogen-bonded to HOH701 and HOH678. The interfacial water molecule is also conserved in HSPCP, in RFPCP reconstituted with different pigments and in the N89L-mutant, where the mutation of Asn-89 to Leu in the vicinity of Per614 has been introduced to study the effects of the environment on the spectroscopic properties [1].

The TR-EPR study was extended to the HPPCP complex whose structural solution is still in progress [102]. The close similarities in terms of the spin-polarization pattern of the triplet state EPR spectra between the PCP complexes of the two different organisms ruled out the possibility of different TTET pathways or that there is a rearrangement of the donor/acceptor pair in HPPCP.

The assumption of spin conservation was verified in PCP complexes reconstituted with ZnChl *a *and BChl* a.* A variation of the donor triplet-state properties in terms of population probabilities (ZnChl *a*) [86] or triplet spin axes directions (BChl* a*) [100] in the reconstituted complexes, was unambiguously reflected in the polarization pattern of the carotenoid triplet state as expected in the framework of spin angular momentum conservation. These results were used to validate the correctness of the spectroscopic approach adopted in the characterization of the TTET pathways in antenna complexes and confirmed that Per 614/624 is the photoprotective carotenoid in all variants of PCP.

Since the distance requirement for TTET is fulfilled for all four Per molecules, other factors, based on fine-tuning of the electron density distribution on the extended conjugated systems and/or the presence of the bridge molecule at the interface between the pair of photoprotective pigments, have been evoked to explain the TR-EPR findings. The role of these factors was addressed by advanced pulse EPR techniques interfaced to laser excitation and combined to DFT calculations of the corresponding spectroscopic observables [102-104].

### 7.4. Pulsed ENDOR and the Electronic Structure of ^T^Per

ENDOR spectroscopy can provide detailed information on the electronic structure of the triplet state, since it permits the determination of the hyperfine coupling (hfc) tensors of the magnetic nuclei interacting with the unpaired electron spins. Chl and Car triplet states possess a large anisotropy of the ZFS tensor, which can be investigated by orientation-selective ENDOR [94].

Porphyrin and (B)Chl model systems [105-108], photosynthetic primary donors [109-112], and carotenoid species populated by TTET in antennae systems [103], have been extensively investigated by pulse ENDOR spectroscopy in order to derive the spin density distribution of their triplet state. Hfcs, reflecting the electron spin density at the magnetic nuclei in the molecule, are closely related to the electronic distribution of the unpaired electrons in the frontier orbitals of the parent singlet ground state. Comparison of the experimental hyperfine parameters with those predicted by reliable Quantum Mechanical computations, allows a comprehensive and detailed description of the electronic structure of the carotenoid triplet state. As already pointed out, the electronic coupling V_TT_ determining the rate in triplet transfer is a four-orbital two-electron exchange integral involving the HOMO and LUMO orbitals. 

Pulse ENDOR experiments of the photo^ T^Per in MFPCP from *A. Cartarae *[103] provided an almost complete set of hyperfine tensor values for all three canonical orientations corresponding to the majority of the protons in the conjugated part of the Per. The ENDOR study was completed with a comparative investigation on MFPCP, HSPCP from *A. carterae, *and HPPCP [102]. No significant differences in hfcs and consequently in triplet spin density on the Per molecules have been detected in PCP complexes from different organisms, with the exception for the variant HSPCP. The discrepancy has been explained in terms of local distortion of the tails of the conjugated chain in the two PCP forms, in agreement with X-ray structural data. The invariance of the ^T^Per hfcs demonstrates that the differences in pigment arrangement between the N-terminal and C-terminal domains, at least in the photoprotection site, are not affecting the triplet state electronic distribution.

**Fig. (8):**
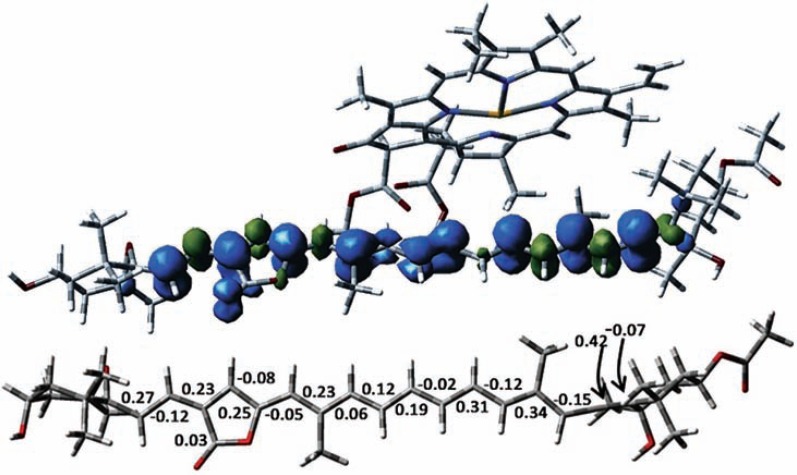
From top to bottom: Computed spin densities for the excited triplet state of Per614 in the Chl601/Per614. Mulliken spin densities computed for the triplet state of Per614. All atoms are at the experimental X-ray geometry, except for methyl groups, allene moiety and hydrogen atoms Only densities larger than 0.02 (in absolute value) are reported. The figure is based on the data from [103].

The isotropic hyperfine constants were assigned by complementing the ENDOR study with DFT calculations. DFT calculations have been performed on the whole Chl-Per complex involved in TTET, considering the Chl601(602)/ Per614(624) couple. This led to the identification of a low-lying triplet state localized on the Per moiety. DFT calculations showed that . the triplet spin density is delocalized over the whole π-conjugated system, with positive spin densities alternating with gradually smaller negative spin densities. This alternate pattern is lost in the central region of the polyene chain where only positive spin densities are found (see Fig. **8**). 

A Q-band ENDOR and DFT study was also performed on the ^T^Per in the refolded N-domain PCP complex (RFPCP). Spectroscopic features of MFPCP are maintained in the RFPCP complex as reported in [112]. 

From the ENDOR studies on PCPs, it was concluded that on the ENDOR time scale the triplet is completely localized on one Per molecule. All the experimental hfcs have been assigned to protons belonging to the Per molecule, no ^T^Chl signals were detected and no reduction of the carotenoid hfcs was observed as would be expected in the case of triplet sharing. The number of identified protons approximately equals the number of protons in the conjugated part of the Per, which confirmed that the triplet is localized on one specific Per molecule at the low temperatures of the spectroscopic experiments (10 K-150 K). Moreover, there was no indication of any delocalization of a triplet between Per and Chl *a* from DFT calculations performed on the triplet state of the Chl-Per complex. Instead, the calculations demonstrated two distinct low-lying triplet states, one on the Chl *a* and the other on the Per [103]. 

### 7.5. Comparison Between Pulsed ENDOR and Step-Scan FTIR Results

As mentioned above, a model based on triplet delocalization between Per and Chl *a* has been used to interpret time-resolved step-scan FTIR difference spectroscopy data from *A. carterae* MFPCP and from HPPCP ([29, 31]; see also [92]). According to this model, the triplet wavefunction is shared between Per and the adjacent Chl. It was proposed that lowering the energy of the shared triplet below that of singlet oxygen, might provide a novel photoprotective mechanism in all oxygenic photosynthetic organisms. 

The ENDOR results point in a different direction and do not provide any evidence of triplet sharing. DFT calculations confirm the ENDOR outcome [103]. The ENDOR evidence of the monomeric nature of the photoprotective triplet state is straightforwardly based on the measured and assigned hfcs of the observed triplet state. Assignment of ENDOR lines to a triplet state shared between the two pigments requires not only the presence of hfcs from both molecules but above all evidence of decrease of the coupling compared to corresponding value for the localized triplet state, as shown for the primary donor in bacterial reaction centers where reduced hfcs from both dimer halves were detected in the triplet state [110]. ENDOR spectroscopy has been extensively used to study electron delocalization in photosynthetic primary donors in different organisms. The monomeric or dimeric nature of the primary donor, the influence of point-mutations in its surroundings on the excitation sharing, could be assessed by mapping the distribution of the unpaired electrons in the molecular system [113]. 

As stated by Alexandre and van Grondelle [92] it seems likely that in the step-scan FTIR experiments at 298 K, the main Per conformer characterized by a lactonic signal at 1745 cm^-1^ corresponds to Per 614, Per 624, or both. It is interesting to note that almost the same value was observed for step-scan FTIR experiments at 100 K [31]; however this value is not in agreement with the one calculated for Per 614 and Per 624 ([22]; see also section 6.2). Therefore, while converging on the interpretation that in MFPCP and HPPCP, Per 614 and/or 624 likely constitute the principal ^T^Chl *a* quenchers, van Grondelle and coworkers assert that aspecific interaction with Chl *a* promotes the mixing of the ^T^Chl *a* and ^T^Per states [92]. 

At this point, a few remarks on the comparability between step-scan FTIR and EPR results should be made, as well as on some general considerations on step-scan FTIR difference spectroscopy (and, more broadly speaking, on differential FTIR spectroscopy).

- As observed in [92] the protein concentration in FTIR samples is generally much higher than in optical and EPR samples and it has been suggested that this could be the origin of the slow decay component observed in 298 K spectra on MFPCP [92].
- Step-scan differential FTIR is a technique where spectra are reconstructed from a series of experimental data and a deep process of data treatment is needed. The danger of artefacts is serious and should not be underestimated. Siebert and coworkers examined in details this issue in detail, pinpointing several possible sources of error ([114-116]; see also [117], from another research group) among which were unwanted heating effect [116]. Indeed, Mezzetti and Spezia [31] showed that some spectral features in the step-scan FTIR spectra from MFPCP from *Amphidinium carterae* [29, 31] are simply a photothermal effect caused by a too high laser power, as reported previously for bacteriorhodopsin [116].
- Identification of bands from pigments is a difficult task in FTIR difference spectroscopy (static or time-resolved) applied to photosynthetic proteins, as almost all the constituents of the proteins absorb in the infrared. In principle any chemical moiety in the protein may vary its chemical structure, its position, conformation or can be in some way “perturbed” as a consequence of the process under study (in this case, light absorption leading to triplet formation) [118]. Therefore assignment of a band in the difference spectrum to a given molecular group must rely on a precise attribution strategy. The most appropriate strategy is the comparison with data on the same protein obtained with isotope-labelled pigments (only pigment bands will show a shift) [118]. If this is not possible, an alternative strategy [91, 119] is to compare difference spectra corresponding to two separate reactions (e.g., formation of a triplet state on the pigment and oxidation of the same pigment). Only bands that coincide will belong to the pigment. If these two strategies cannot be applied, only tentative assignments are possible. However if several lines of evidence are combined a tentative assignment may be quite robust. In the case of the 1745 cm^-1^ (at 298 K) or 1746 cm^-1^ (at 100 K) negative band, Mezzetti and Spezia assigned it to the lactonic C=O of Per based on 1) comparison with FTIR spectra of isolated Per [120]; 2) decay kinetics of the band in agreement with time-resolved UV-Vis data [74]; 3) DFT calculations on Per and ^T^Per [31]; 4) simultaneous presence of other bands belonging to Per ([31]; see also [22]); 5) comparison with previously reported triplet minus singlet FTIR spectra in photosynthetic proteins [91, 119]. Alexandre *et al.* [29] assigned the bands at 1699 and 1686 cm^-1^ in their spectra to Chl *a* 9-keto by: 1) comparing it to FTIR spectra of Chl *a* in solution [120, 121]; 2) by comparing it to Fluorescence Line Narrowing data of PCP at 4 K [74]; and 3) on the fact that positive bands are observed in a position where ^T^Chl *a* vibrations lie [121]. However, this plausible assignment should be considered as tentative because a) the 1699 and 1686 cm^-1^ negative bands are not observed at 100 K [31]; b) the region considered for Chl *a* and ^T^Chl *a* 9-keto bands is also the region where the amide I (protein backbone) and several other residues absorbs; c) the presence of the 9-keto Chl *a* band as a negative feature can be also explained as a modification of the 9-keto environment induced by triplet formation. In addition, the 1690-1610 cm^-1^ region is much noisier than other spectral regions. Data analysis in this range should always be taken with care, unless an excellent signal-to-noise ratio is achieved.
- Step-scan FTIR spectroscopy has a mandatory requirement: the reaction under study should be perfectly reproducible for several thousand cycles [115]. This can be critical when dealing with low signals such as those originating from triplet formation that need long accumulation times. A common strategy [31, 90, 91] is to record step-scan FTIR spectra at low T to preserve the sample integrity as long as possible.
- The step-scan FTIR data treatment procedure deserves a final comment. Van Grondelle and coworkers use global and target analysis, which rely on the *a priori* formulation of a kinetic model [92]. Many authors prefer to use simpler single wavenumber kinetics (e.g. [31, 90, 122]), or alternative analytical strategies, such as multivariate curve resolution ([123, 124], and refs therein) or 2D correlation spectroscopy [124]. These techniques do not rely on the formulation of any kinetic model.

### 7.6. Theoretical Calculations on the Electronic Structure of ^T^Per

The electronic structure of Per, and carotenoids in general, has proved extremely challenging to calculate. While the excited singlet state was explored in detail by DFT methodologies (see section 2), the information on the triplet state is still incomplete for Per and this class of molecules, whose biological relevance is fundamental. The use of pulse ENDOR spectroscopy combined with DFT calculations has illuminated the triplet state of the Per, by mapping the spin and electron densities, in terms of frontier orbitals, over the whole conjugated system. The satisfactory agreement between the experimental and computed hfcs has provided a critical test of the triplet state wavefunction, which is not mixed with higher-energy excitations and is characterized by a predominant HOMO→LUMO character.

The outcome of the DFT calculations is that the triplet-state electron density is almost equally distributed all over the entire π-electron conjugated system and is not perturbed by the presence of molecular substituents. A comparison of carotenoid triplet spin density distribution in Per and in the symmetric carotenoid, lutein, revealed no significant differences [125]. The presence in the Per molecule of an allene moiety and a lactone ring, both of which are connected directly to the π-system, does not significantly alter the spin density pattern.

As already pointed out in Section 2, the singlet excited state behaviour of Per is strongly influenced by the carbonyl group forming part of the π-electron conjugated chain. The marked spectral differences between Per and carbonyl-containing carotenoids and symmetric carotenoids are not preserved in the triplet state properties. In a transient absorption study of the triplet state of a series of Per analogues *in vitro*, it was demonstrated that Per in the triplet state is insensitive to solvent polarity and that no low-lying intramolecular charge-transfer state is present in the triplet manifold [126]. 

### 7.7. TTET Electronic Coupling and the Superexchange Mechanism 

An extended and well-distributed electron density allows structural flexibility in the arrangement of the carotenoid-chlorophyll pairs in the protein scaffold of different light-harvesting complexes and also represents an important requisite for pigments involved in light-harvesting and structural functions. Due to the large dimensions of the carotenoid and Chl *a* macrocycle, a close center-to-center distance between donor and acceptor allows the involvement of an extended part of the polyene in the TTET electron coupling. The widespread distribution of the electron density is therefore required in order to optimize the wavefunction overlap. Since the broad electronic distribution does not preclude an adequate overlap of any of the four Pers in van der Waals contact with the Chl *a* macrocycle in PCP, it was suggested that the molecular bridge might be the structural element responsible for the selection of the specific Per-Chl *a* pair involved in TTET. 

The superexchange model originally developed by McConnell for electron transfer [127] was later extended to TTET [83]. 

The interaction of an intervening water molecule with ^T^Per in the PCP complex from *A. carterae* was studied by using orientation selective ^2^H electron spin echo envelope modulation (ESEEM), in conjunction with quantum mechanical calculations [104]. 

ESEEM provides information on small proton hyperfine couplings, due to nuclei that are not bound to the paramagnetic centre directly and that are not resolved in the ENDOR spectra [128, 129]. ESEEM can be combined with hydrogen-deuterium exchange in order to investigate selectively the exchangeable water protons in proximity to the paramagnetic center. ESEEM experiments on the ^2^H_2_O-exchanged MFPCP complex from *A. cartarae* have allowed a detailed characterization of the photoprotective site [104]. Simulations of the ESEEM experiments demonstrated the interaction of two coupled ^2^H belonging to HOH701/HOH678. The involvement of the water bridge in the TTET process, which in PCP occurs with unit efficiency, is an important model for future investigations.

Theoretical methods to characterise the TTET coupling [130] are currently not as well developed as for electron transfer couplings. In the specific case of the PCP complex, computational studies, based on different approaches, have provided estimates of the electronic coupling matrix elements for all Chl *a*/Per pairs. Damjanović *et al.* estimated, by direct calculation of the exchange integrals using a semiempircal model, that the largest exchange coupling involved the Chl601–Per613 pair [79], in contrast to the EPR spectroscopic evidence [98]. Prompted by the EPR investigations on the PCP complex, the effect of the water molecule coordinated to chlorophyll on the TTET coupling has also been considered [130, 131]. A previously developed method in the *ab initio* framework [130], was employed for the calculation of the TTET electronic coupling between pigments in PCP. The Chl602-Per624 pair gave the largest coupling among the four Pers contained in one pigment cluster, in agreement with the EPR results. However, the bridging water molecule made a negative contribution to the coupling, which was attributed to an opposite phase of the water-mediated coupling with respect to the through-space coupling. Surprisingly, the same water molecule was found to increase the coupling between Chl602 and another Per molecule, Per623, positioned on the opposite side with respect to the chlorophyll ring. In a previous study, based on the MP2 perturbation method, on π-π stacking interactions between pigments in the PCP complex, the same water molecule, at the interface between Chl602 and Per624, was found to give a significant contribution to the intermolecular interaction energy between the two pigments [73], in agreement with EPR results.

The double exchange integral plays a key role in determining the rate constants of the TTET processes and their accurate prediction is an important issue. The lack of consensus on the estimate of the TTET couplings derives from the origin of this small electronic interaction involving the tails of the wavefunctions. For this reason an accurate description of the frontier orbital involved, also considering superexchange contributions from bridging molecules in detail, is an absolute requirement for future calculations. Advanced EPR spectroscopy on ^T^Per in PCP may give an important contribution to this issue. 

## 8. TECHNOLOGICAL APPLICATIONS

The peculiar photophysical properties of PCPs and Per has suggested their use in a “technological” framework.

### 8.1. PCPs Coupled to Nanostructures

The coupling of PCP proteins with inorganic nanostructures can lead to strong enhancement of both the absorption and of the fluorescence of the protein complex [10, 132-139].

PCPs on silver island films [132], semicontinuous silver films [133]; spherical gold nanoparticles [10, 134], silver nanowires [135] silica nanoparticles [136] have been reported. The topic has been recently reviewed [137, 138] so here we just underline its strong potential in a technological perspective, both in the field of hybrid nanostructures for light energy conversion, and as a tool for emission enhancement of PCPs as a fluorophore.

### 8.2. Artificial Light-Harvesting Complexes

Carotenoids have been largely used in molecular complexes for artificial photosynthesis, especially for light harvesting purposes. Per, as other carbonyl containing carotenoids, presents some intriguing properties in artificial antennae such as 1) shrinking of the S_2_-S_1_ energy gap, which means that there are able to harvest light in the 450-550 nm range, but keeping their S_1_ state high enough to transfer energy efficiently to the Q_Y_ band of porphyrin-like acceptors; 2) excited-state properties can be tuned by changing solvent polarity (see also paragraph 2).

A Per-pyropheophorbide dyad was synthesized and investigated in the late 90s [140, 141], before the discovery of the peculiarity of Per photophysical properties. As a consequence, only limited information could be obtained, i.e. that the energy transfer from Per to pyropheophorbide and that Per is able to quench the triplet states of the pyropheophorbide moiety (mimicking therefore the photoprotective role of carotenoids in natural systems) [141]. A later investigation [142] on the same dyad demonstrated the possibility of tuning the energy transfer from Per to the pyropheophorbide moiety by changing the polarity of the solvent. It is interesting that the energy transfer efficiency observed in this dyad is much higher compared to that observed for the analogue dyad where Per is replaced by fucoxanthin, underlying the high potential interest in Per for the development of artificial antennae.

### 8.3. PCP as a Fluorophore in Biomedical Research

The high quantum yield of fluorescence of PCP, along with the large stokes shift observed between absorption and fluorescence bands, has suggested the use of PCP as a fluorescent label in diagnostic assays [143]. Since then, PCPs have been widely used as fluorophores in biomedical studies, see for instance [9; 144,^ 145^]. It is interesting to note that this particular application started between the end of the 80s and the early 90s, long before the beginning of the detailed photophysical characterization of PCPs described in this review. It should finally be mentioned that recent research has suggested a possible anti-cancer role of Per [146, 147]; physicochemical studies might help to understand this peculiar Per role at a molecular level.

## 9. CONCLUSIONS AND PERSPECTIVES

PCPs represents a very peculiar photophysical system, and represents a model system to investigate energy transfer mechanism in light-harvesting complexes. Furthermore, some technological applications have been reported and research in this domain has been particularly active in the last years.

Several issues remain to be resolved, these are:

1. the exact nature of the ICT state;
2. the mechanism of singlet-singlet energy transfer;
3. a detailed theoretical description of Per electronic states;
4. the mechanism of photoprotection, since precise localization of the triplet state is uncertain;
5. identification of vibrational marker bands to distinguish individual Per in PCPs;
6. the detailed description of how the vibrational properties of Per are tuned by the environment and by the electronic state of the molecule.

In addition, whereas most of the studies have been focused on MFPCP from *A. carterae*, the spectroscopic characterization of RFPCP (including RFPCP reconstituted with different Chls), HSPCP and HP-PCP is still fragmentary. It should also be mentioned that other PCPs have been discovered and – despite the lack of an X-ray structure – their spectroscopic characterization has started. The use of new advanced spectroscopic techniques may also bring new insight into the energy transfer in PCP (for instance, single-molecule studies have been reported [148]). Other established techniques such as Resonance Raman spectroscopy have not been widely used yet, although more than 30 years ago Resonance Raman Micro-spectroscopy was used to visualize Per in living cells [69, 70]. The coupling between PCPs and nanostructures to modulate PCP photophysics and the development of artificial antennae containing Per as the light harvesting pigment are also a very promising field of research. 

## Figures and Tables

**Table 1.** Summary of Per vibrational modes between 1500 and 2000 cm^-1^. Abbreviations: PMS = peridinin model system; ACN=acetonitrile; CHX = cyclohexane; MeOH = methanol.

Infrared (solid state) [22]	Raman (solid state) [28]	Calculations in vacuum [28]	MD (on PMS) (vacuum/CHX/ACN/MeOH) [22]	Resonance Raman in solvents(CHX/ACN/MeOH) [22]	Assignment
1925	1928	1934	1953/1963/ 1952/1955	1929/ 1929/1929	Allene [28, 22]
1740, 1717 (sh), 1771 (sh)	1747	1751	1762/1748/ 1717/1709	1765-1777/ 1751/1736-1756	stretching of the C=O of the lactonic ring. The 1717 cm-1 band observed in IR was assigned to the stretching of the ester C=O [22]
1643			1621/1624/ 1623/1624		Polyene chain [22]
1622	1616	1610	1601/1597/ 1598/1597	1614/ 1616/1614	Polyene chain [22]
1596			1582/1578/ 1577/1579		Polyene chain [22]
	1590	1587	1575/1574/ 1575/1570	-/ 1589/1588	Polyene chain [22]
1538	1540	1518	1538/1531/ 1527/1526	1563/ 1565/1568	Polyene chain [22]
1521	1522	1503	1517/1516/ 1513/1512	1540/ 1540/1538	Polyene chain [22]
			1496/1492/ 1494/1489	1525/ 1526/1524	Polyene chain [22]

**Table 2.** Summary of Per low lying electronic excited states. Abbreviation: 2-MTHF= 2-methyltetrahydrofuran. Values are given in wave numbers or in nm (in brackets).

	S1	S2		(S3)	Notes
TDDFT/TDA [26]	18224 (549) Ag	19461 (514) Bu		21360 (468) ICT	Full optimized structure
TDDFT/TDA [26]	16394 (610) Ag	18597 (538) ICT		19211 (521) Bu	From Per 611 structure
MNDO-PSDCI [25]	15809 (633) Ag-	19277 (519) Bu+		22584 (443) Bu-	
TD-SVWN [27]	16696 (599)	17986 (556)			
Experimental [16]		0-0 0-1 0-2	19060 (525) 19980 (501) 20120 (497) 20550 (487) 21390 (467) 21730 (460) 22070 (453)		In 2-MTHF

## References

[1] Schulte T, Johanning S, Hofmann E (2010). Structure and function of native and refolded peridinin-chlorophyll-proteins from dinoflagellates.. Eur. J. Cell Biol..

[2] Polívka T, Hiller RG, Frank HA (2007). Spectroscopy of the peridinin-chlorophyll-a protein insight into light-harvesting strategy of marine algae.. Arch. Biochem. Biophys..

[3] Song PS, Koka P, Prezelin BB, Haxo FT (1976). Molecular topology of photosynthetic light-harvesting pigment complex, peridinin-chlorophyll-a-protein, from marine dinoflagellates.. Biochemistry.

[4] Koka P, Song PS (1977). The chromophore topography and binding environment of peridinin-chlorophyll-a-protein complexes from marine dinoflagellate algae Biochim.. Biophys. Acta General Subjects..

[5] Hofmann E, Wrench PM, Sharples FP, Hiller RG, Welte W, Diederichs K (1996). Structural basis of light harvesting by carotenoids peridinin-chlorophyll-protein from Amphidinium carterae.. Science.

[6] Schulte T, Niedzwiedzki DM, Birge RR, Hiller RG, Polivka T, Hofmann E, Frank HA (2009). Identification of a single peridinin sensing Chl-a exctitation in reconstituted PCP by crystallography and spectroscopy.. Proc. Natl. Acad. Sci. U.S.A..

[7] Schulte T, Sharples FP, Hiller RG, Hofmann E (2009). X-ray structure of the high-salt form of peridinin-chlorophyll-a protein from the Dinoflagellate Amphidinium carterae modulation of the spectral properties of pigments by the protein environment.. Biochemistry.

[8] Schulte T, Hiller RG, Hofmann E (2010). X-ray structures of the peridinin-chlorophyll-proteins reconstituted with different chlorophylls.. FEBS Lett..

[9] Niki S, Hosokawa K, Nagaike K, Tagawa T (2004). A new immunofluorostaining method using red fluorescence of PerCP of formalin-fixed paraffin-embedded tissues J.. Immunol. Methods.

[10] Olejnik M, Krajnik B, Kowalska D, Lin G, Mackowski S (2013). Spectroscopic studies of plasmon coupling between photosynthetic complexes and metallic quantum dots.. J. Phys. Condens. Matter.

[11] Frank HA, Young AJ, Britton G, Cogdell RJ, Christensen RL (1999). The electronic states of carotenoids in The photochemistry of carotenoids.. Kluwer Academic Publishers..

[12] Polivka T, Sundstrom V (2004). Ultrafast dynamics of carotenoid excited States-from solution to natural and artificial systems.. Chem Rev..

[13] Mimuro M, Nagashima M, Takaichi S, Nishimura Y, Yamazaki I, Katoh T (1992). Molecular structure and optical properties of carotenoids for the in vivo energy transfer function in algal photosynthetic pigment system.. Biochim. Biophys. Acta Bioenergetics..

[14] Bautista JA, Connors RE, Raju BB, Hiller RG, Sharples FP, Gostzola D, Wasielewski MR (1999). Excited state properties of peridinin observation of a solvent dependence of the lowest excited singlet state lifetime and spectral behavior unique among carotenoids.. J. Phys. Chem. B.

[15] Kajikawa T, Katsumu S (2012). An Approach Based on Synthetic Organic Chemistry Toward Elucidation of Highly Efficient Energy Transfer Ability of Peridinin in Photosynthesis in Artificial photosynthesis Mohammad Mahdi Najafpour.. Intechopen..

[16] Niedzwiedzki DM, Kajikawa T, Aoki K, Katsumura S, Frank HA (2013). Excited states energy and dynamics of peridinin analogues and tyhe nature of the intramolecular charge ttranfer state in carbonyl-containing carotenoids, J.. Phys. Chem. B.

[17] Enriquez MM, Hananoki S, Hasegawa S, Kajikawa T, Katsumura S, Wagner NL, Birge RR, Frank HA (2012). Effect of molecular symmetry on the spectra and dynamics of Intramolacular Charge Transfer state of Peridinin, J.. Phys. Chem. B..

[18] Kusumoto T, Horibe T, Kajikawa T, Hasegawa S, Iwashita T, Cogdell RJ, Birge RR, Frank HA, Katsumura S, Hashimoto H (2010). Stark absorption spectroscopy of peridinin and allene-modified analogues.. Chem. Phys..

[19] Niedzwiedzki DM, Chattarjee N, Enriquez MM, Kajikawa T, Hasegawa S, Katsumura S, Frank HA (2009). Spectroscopic investigation of peridinin analogues having different ?-electron conjugated chain length, exploring the nature of the intramolecular charge transfer state, J.. Phys. Chem. B.

[20] Kajikawa T, Aoki K, Iwashita T, Niedzwiedzki DM, Frank HA, Katsumura S (2010). Syntheses of ylidenbutenolide-modicfied derivatives of peridinin and their stereochemical and spectral characteristics.. Org. Biomolec. Chem..

[21] Kajikawa T, Hasegawa S, Iwashita T, Kusumoto T, Hashimoto H, Niedzwiedzki DM, Frank HA, Katsumura S (2009). Syntheses of C33 C35 and C39-peridinin and their spectral characteristics.. Org Lett..

[22] Bovi D, Mezzetti A, Vuilleumier R, Gaigeot MP, Chazallon B, Spezia R, Guidoni L (2011). Environmental effects on vibrational properties of carotenoids experiments and calculations on peridinin, Phys.. Chem. Chem. Phys..

[23] Van Tassle AJ, Prantil MA, Hiller RG, Fleming GR (2007). Excited state structural dynamics of the charge transfer of peridinin.. Isr. J. Chem..

[24] Bonetti C, Alexandre MTA, van Stokkum IHM, Hiller RG, Groot ML, van Grondelle R, Kennis JTM (2010). Identification of excited-state energy transfer and relaxation pathways in the peridinin-chlorophyll complex an ultrafast mid-infrared study.. Phys. Chem. Chem. Phys..

[25] Shima S, Ilagan RP, Gillespie N, Sommer BJ, Hiller RG, Sharples FP, Frank HA, Birge RR (2003). Two-photon and fluorescence spectroscopy and the effect of environment on the photochemical properties of peridinin in solution and in the Peridinin-Chlorophyll-Protein from Amphidinium carterae.. J. Phys. Chem. A.

[26] Vaswani HM, Hsu CP, Head-Gordon M, Fleming GR (2003). Quantum chemical evidence for an intramolecular charge-transfer state in the carotenoid peridinin of Peridinin-Chlorophyll-Protein.. J. Phys. Chem. B.

[27] Spezia R, Zazza C, Palma A, Amadei A, Aschi M (2004). A DFT study of Low-Lying Singlet Excited States of the all-trans Peridinin in vacuo.. J. Phys. Chem. A.

[28] Dietzek B, Chabera P, Hanf R, Tschierlei S, Popp J, Pascher T, Yarsev A, Polivka T (2010). Optimal control of peridinin excited-state dynamics.. Chem. Phys..

[29] Alexandre MTA, Luhrs DC, van Stokkum IHM, Hiller R, Groot ML, Kennis JTM, van Grondelle R (2007). Triplet state dynamics in peridinin-chlorophyll-a-protein: a new pathway of photoprotection in LHCs?.. Biophys. J..

[30] Bonetti C, Alexandre MTA, Hiller RG, Kennis JTM, van Grondelle R (2009). Chl-a triplet quenching by peridinin in H-PCP and organic solvent revealed by step-scan FTIR time-resolved spectroscopy,. Chem.Phys..

[31] Mezzetti A, Spezia R (2008). Time-resolved step scan FTIR spectroscopy and DFT investigation on triplet formation in peridinin-chlorophyll-a-protein from Amphidinium carterae at low temperature. Spectroscopy Int. J.,.

[32] Wagner NL, Greco JA, Enriquez MM, Frank HA, Birge RR (2013). The nature of the intramolecular charge transfer state in peridinin.. Biophys. J..

[33] Frank HA, Bautista JA, Josue J, Pendon Z, Hiller RG, Sharples FP, Goszola D, Wasielewski MR (2000). Effect of the Solvent Environment on the Spectroscopic Properties and Dynamics of the Lowest Excited States of Carotenoids.. J. Phys. Chem. B.

[34] Zigmantas D, Polivka T, Hiller RG, Yartsev A, Sundstrom V (2001). Spectroscopic and Dynamic Properties of the Peridinin Lowest Singlet Excited States.. J. Phys. Chem. A.

[35] Zigmantas D, Hiller RG, Sharples FP, Frank HA, Sundstrom V, Polivka T (2004). Effect of a conjugated carbonyl group on the photophysical properties of carotenoids, Phys.. Chem. Chem. Phys..

[36] Linden PA, Zimmermann J, Brixner T, Holt NE, Vaswani HM, Hiller RG, Fleming GR (2004). Transient absorption study of peridinin and peridinin-chlorophyll a-protein after two-photon excitation.. J. Phys. Chem. B.

[37] Papagiannakis E, Larsen DS, van Stokkum IHM, Vengris M, Hiller RG, van Grondelle R (2004). Resolving the excited state equilibrium of peridinin in solution.. Biochemistry..

[38] Zigmantas D, Hiller RG, Sundstrom V, Polivka T (2002). Carotenoid to chlorophyll energy transfer in the peridinin-chlorophyll-a-protein complex involves an intramolecular charge transfer state.. Proc. Natl. Acad. Sci. U.S.A..

[39] Polivka T, Pascher T, Sundstrom V, Hiller RG (2005). Tuning energy transfer in the peridinin-chlorophyll complex by reconstitution with different chlorophylls.. Photosynth. Res..

[40] Bautista JA, Hiller RG, Sharples FP, Goszola D, Wasielewski M, Frank HA (1999). Singlet and triplet energy transfer in the Peridinin-Chlorophyll a-Protein from Amphidinium carterae.. J. Phys. Chem. A.

[41] Zigmantas D, Hiller RG, Yarsev A, Sundstrom V, Polivka T (2003). Dynamics of exicted states of the carotenoid peridinin in polar solvents dependence on excitation wavelength viscosity and temperature.. J. Phys. Chem. B.

[42] Papagiannakis E, Vengris M, Larsen DS, van Stokkum IHM, Hiller RG, van Grondelle R (2006). Use of ultrafast dispersed pump-dump-probe and pump-repump-probe spectroscopies to explore the light-induced dynamics of peridinin in solution J.. Phys. Chem. B.

[43] König C, Neugebauer J (2012). Quantum chemical description of absorption properties and excited-state processes in photosynthetic systems.. ChemPhysChem..

[44] Martin CH, Birge RR (1998). Reparametrizing MNDO for Excited-State Calculations by Using ab Initio Effective Hamiltonian Theory?Application to the 2-4-Pentadien-1-iminium Cation J.. Phys. Chem. A.

[45] Enriquez MM, Fuciman M, LaFountain AM, Wagner NL, Birge RR, Frank HA (2010). The Intramolecular Charge Transfer State in Carbonyl-Containing Polyenes and Carotenoids J.. Phys. Chem. B.

[46] Krylov AI (2008). The spin-flip equation-of-motion coupled-cluster electronic structure method for a description of excited states, bond-breaking, diradicals and triradicals Ann.. Rev. Phys Chem..

[47] Gouterman M (1961). Spectra of porphyrins.. J. Mol. Spectrosc..

[48] Gouterman M, Wagnière GH, Snyder LC (1963). Spectra of porphyrins Part II Four orbital model.. J. Mol. Spectrosc..

[49] Linnanto J, Korppi-Tommola J (2000). Spectroscopic properties of Mg-chlorin, Mg-porphin and chlorophylls a b c1 c2 c3 and d studied by semiempirical MO/CI methods.. Phys. Chem. Chem. Phys..

[50] Linnanto J, Korppi-Tommola J (2004). Structure and Spectroscopic Properties of Mg-Bacteriochlorin and Methyl Bacteriochlorophyllides a b g and h Studied by Semiempirical Ab Initio and Density Functional Molecular Orbital Methods.. J. Phys. Chem. A.

[51] König C, Neugebauer J (2011). First-Principles Calculation of Light-Harvesting Complex II.. Phys. Chem. Chem. Phys..

[52] Scheer H (1991). Chlorophylls CRC Press.. Boca Raton..

[53] Damjanovic A, Vaswani HM, Fromme P, Fleming GR (2002). Chlorophyll Excitations in Photosystem I of Synechococcus elongates.. J. Phys. Chem. B.

[54] Adolphs J, Müh F, Madjet ME, Renger T (2008). Calculation of pigment transition energies in the FMO protein.. Photosynth. Res..

[55] Adolphs J, Müh F, Madjet ME, Schmidt am Busch MS, Renger T (2010). Structure-based calculations of optical spectra of photosystem I suggest an asymmetric light-harvesting process.. J. Am. Chem. Soc..

[56] Katz JJ, Closs GL, Pennington FC, Thomas MR, Strain HH (1963). Infrared spectra, molecular weights, and molecular association of chlorophylls a and b methyl chlorophyllides and pheophytins in various solvents.. J. Am. Chem. Soc..

[57] Oksanen JAI, Martinsson P, Åkesson E, Hynninen PH, Sundström V (1998). Transient Hole Burning and Solvation Dynamics of Chlorophyll b Monomers in Various Solvent Environments.. J. Phys. Chem. A.

[58] Fiedor L, Kania A, Orzel L, Mysliwa-Kurdziel B, Stochel G (2008). Understanding Chlorophylls Central Magnesium Ion and Phytyl as Structural Determinants.. Biochim. Biophys. Acta Bioenerg..

[59] Giacometti G, Agostini G, Santabarbara S, Carbonera D (2007). ODMR spectroscopy of molecular functions in photosynthetic membrane proteins.. Appl. Magn. Res..

[60] Budil DE, Thurnauer MC (1991). The chlorophyll triplet state as a probe of structure and function in photosynthesis.. Biochim. Biophys. Acta-Bioenerg..

[61] den Blanken HJ, van der Zwet GP, Hoff AJ (1982). ESR in zero field of the photoinduced triplet state in isolated reaction centers of rhodopseudomonas sphaeroides R-26 detected by the singlet ground-state absorbance.. Chem. Phys Lett..

[62] Schaafsma TJ (1982). In Triplet State ODMR Spectroscopy R..

[63] Breton J (2001). Fourier transform infrared spectroscopy of primary electron donors in type I photosynthetic reaction centers Biochim.. Biophys. Acta Bioenerg..

[64] Robert B (2009). Resonance Raman spectroscopy.. Photosynth. Res..

[65] Carbonera D, Giacometti G, Segre U (1996). Carotenoid interactions in Peridinin Chlorophyll a Proteins from Dinoflagellates evidence for optical excitons and triplet migration J.. Chem. Soc. Far. Trans..

[66] Niedzwiedzki DM, Jiang J, Lo CS, Blankenship RE (2013). Low-Temperature Spectroscopic Properties of the Peridinin–Chlorophyll a–Protein (PCP) Complex from the Coral Symbi-otic Dinoflagellate Symbiodinium.. J. Phys. Chem. B.

[67] Ogata T , Kodama M, Nomura S, Kobayashi M, Nozawa T, Katoh T, Mimuro M (1994). A novel peridinin-chlorophyll a protein (PCP) from the marine dinoflagellate Alexandrium cohorticula a high pigment content and plural spectral forms of peridinin and chlorophyll a.. FEBS Lett..

[68] Sharples FP, Wrench PM, Ou K, Hiller RG (1996). Two distinct forms of the peridinin-chlorophyll a-protein from Amphidinium carterae.. Biochim. Biophys. Acta Bioenergetics..

[69] Merlin JC (1983). La microspectrométrie Raman de Résonance méthode d’investigation in vivo des systèmes pigmentaires.. Spectrosc. Int. J..

[70] Dupaix A, Arrio B, Lecuyer B, Fresneau C, Merlin JC (1982). Intracellular Sepctroscopic studies of a bioluminescent cell.. Pyrocystis lunula. Biol Cell..

[71] Miller DJ, Catmull J, Puskeiler R, Tweedale H, Sharples FP, Hiller RG (2005). Reconstitution of the peridinin-chlorophyll-a protein (PCP) evidence for functional flexibility in chlorophyll binding.. Photosynth Res..

[72] Spezia R, Aschi M, Di Nola A, Di Valentin M, Carbonera D, Amadei A (2003). The effect of protein confor-mational flexibility on the electronic properties of a chromophore.. Biophys. J..

[73] Mao L, Wang Y, Hu X (2003). p-p stacking interactions in the peridinin-chlorophyll-protein of Amphidinium carterae.. J. Phys. Chem. B.

[74] Kleima FJ, Wendling M, Hofmann H, Peterman EJG, van Grondelle R, van Amerongen H (2000). Peridinin chlorophyll a protein relating structure and steady state spectroscopy.. Biochemistry..

[75] Carbonera D, Giacometti G, Segre U, Hofmann E, Hiller RG (1999). Structure-based calculations of the optical spectra of the ligth-harvesting peridin-chlorophyll-protein complexes from Amphydinium carterae and Heterocapsa pygmaea.. J. Phys. Chem. B.

[76] Ilegan RP, Shima S, Melkozernov A, Lin S, Blankenship RE, Sharples FP, Hiller RG, Birge RR, Frank HA (2004). Spectroscopic properties of the main-form and high-salt peridinin-chlorophyll a proteins from Amphidinium carterae.. Biochemistry..

[77] Krueger BP, Lampoura SS, van Stokkum IHM, Papagiannakis E, Salverda JM, Gradinaru CC, Rutkauskas D, Hiller RG, van Grondelle R (2001). Energy transfer in the peridinin chlorophyll-a protein of Amphidinium carterae studied by polarized transient absorption and target analysis.. Biophys. J..

[78] Akimoto S, Takaichi S, Ogata T, Nishimura Y, Yamazaki I, Mimuro M (1996). Excitation energy transfer in carotenoid-chlorophyll protein complexes probed by femtosecond fluorescence decays.. Chem. Phys. Lett..

[79] Damjanovic A, Ritz T, Schulten K (2000). Excitation transfer in the Peridinin-Chlorophyll-Protein of Amphidinium carterae.. Biophys. J..

[80] Frank HA, Brudvig GW (2004). Redox functions of carotenoids in photosynthesis.. Biochemistry.

[81] Young A, Britton G (1993). Carotenoids in photosynthesis..

[82] Dexter DLJ (1953). A theory of sensitized luminescence in solids.. Chem. Phys..

[83] Closs GI, Johnson MD, Miller JR, Piotrowiak P (1989). A connection between intramolecular long-range electron hole and triplet energy transfers.. J. Am. Chem. Soc..

[84] Marcus RA (1964). Chemical and Electrochemical electron Transfer Theory.. Ann. Rev. Phys. Chem..

[85] Carbonera D, Giacometti G, Agostini G (1995). FDMR spectroscopy of peridinin-chlorophyll-a protein from Amphidinium carterae.. Spectrochim. Acta Part A..

[86] Clarke RH, Chan IY (1982). In ODMR spectroscopy Techniques and Applications to Biological Systems.. ..

[87] Hoff AJ (1989). In Advanced EPR.. Elsevier Amsterdam..

[88] Carbonera D (2009). Optically detected magnetic resonance (ODMR) of photoexcited triplet states Photosynth.. Res..

[89] Carbonera D, Agostini G, Morosinotto T, Bassi R (2005). Quenching of chlorophyll triplet states by carotenoids in reconstituted Lhca4 subunit of peripheral light-harvesting complex of photosystem I.. Biochemistry..

[90] Burie JR, Leibl W, Nabedryk E, Breton J (1993). Step-scan FT-IR spectroscopy of electron transfer in the photosynthetic bacterial reaction center.. Appl. Spectr..

[91] Mezzetti A, Seo D, Leibl W, Sakurai H, Breton J (2003). Time-resolved step-scan FTIR investigation on the primary donor of the reaction center from the green sulfur bacterium Chlorobium tepidum.. Photosynth. Res..

[92] Alexandre M, van Grondelle R (2012). Time-Resolved FTIR Difference Spectroscopy Reveals the Structure and Dynamics of Carotenoid and Chlorophyll Triplets in Photosynthetic Light-Harvesting Complexes in Infrared Spectroscopy Life and Biomedical Sciences Theophanides.. In Techopen..

[93] Moebius K, Lubitz W, Savitsky A (2011). Photo-Induced Electron Spin Polarization in Chemical and Biological Reactions: Probing Structure and Dynamics of Transient Intermediates by Multifrequency EPR Spectroscopy.. Appl. Mag. Res..

[94] Lubitz W (2002). Pulse EPR and ENDOR studies of light-induced radicals and triplet states in photosystem II of oxygenic photosynthesis.. Phys. Chem. Chem. Phys..

[95] El-Sayed MA (1971). Optical Pumping of the Lowest Triplet State and Multiple Resonance Optical Techniques in Zero Field.. J. Chem. Phys..

[96] Brenner HC, Brock JC, Harris CB (1978). Energy exchange in a coherently coupled ensemble.. Chem. Phys..

[97] Di Valentin M, Tait C, Salvadori E, Ceola S, Scheer H, Hiller RG, Carbonera D (2011). Conservation of Spin Polarization during Triplet-Triplet Energy Transfer in Reconstituted Peridinin-Chlorophyll-Protein Complexes.. J. Phys. Chem. B.

[98] Di Valentin M, Ceola S, Salvadori E, Agostini G, Carbonera D (2008). Identification by time-resolved EPR of the peridinins directly involved in chlorophyll triplet quenching in the peridinin-chlorophyll a-protein from Amphidinium carterae.. Biochim. Biophys. Acta Bioenerg..

[99] Angerhofer A (1991). Chlorophylls CRC Press.. Florida..

[100] Vrieze J, Hoff AJ (1995). The orientation of the triplet axes with respect to the optical transition moments in (bacterio) chlorophylls.. Chem. Phys. Lett..

[101] Frick J, Von Schutz JU, Wolf HC, Kothe G (1990). First detection of the (nonphosphorescent) triplet state in single crystals of ?-carotene.. Mol. Liq. Cryst. Liq. Cryst..

[102] Di Valentin M, Salvadori E, Ceola S, Carbonera D (2010). Pulsed EPR and ENDOR on the Peridinin Triplet State Involved in the Photoprotective Mechanism in Peridinin-Chlorophyll a-Proteins.. Appl. Mag. Res..

[103] Di Valentin M, Ceola S, Agostini G, Giacometti GM, Angerhofer A, Crescenzo O, Barone V, Carbonera D (2008). Pulse ENDOR and density functional theory on the peridinin triplet state involved in the photo-protective mechanism in the peridinin-chlorophyll a-protein from Amphidinium carterae.. Biochim. Biophys. Acta Bioenerg..

[104] Di Valentin M, Tait CE, Salvadori E, Orian L, Polimeno A, Carbonera D (2014). Evidence for water-mediated triplet–triplet energy transfer in the photoprotective site of the peridinin–chlorophyll a–protein.. Biochim. Biophys. Acta Bioenerg..

[105] Kay CWM, Di Valentin M, Mobius K (1995). A time-resolved Electron Nuclear Double Resonance (ENDOR) study of the photoexcited triplet state of free-base tetraphenylporphyrin.. Sol. Energy Mater. Sol. Cells.

[106] Kay CWM, Di Valentin M, Mobius K (1997). Time-resolved EPR and ENDOR study of the photoexcited triplet state of free-base tetraphenylchlorin in a crystalline toluene matrix.. J. Chem. Soc. Perkin Trans II..

[107] Kay CWM, Gromadecki U, Törring JT, Weber S (2001). An investigation of the structure of free-base porphycene by time-resolved electron nuclear double resonance and density functional theory on the photoexcited triplet state.. Mol. Phys..

[108] Marchanka A, Lubitz W, van Gastel M (2009). Spin Density Distribution of the Excited Triplet State of Bacteri-ochlorophylls.Pulsed ENDOR and DFT Studies.. J. Phys. Chem. B.

[109] Di Valentin M, Kay CWM, Giacometti G, Mobius K (1996). A time resolved Electron Nuclear Double Resonance study of the Photoexcited Triplet State of P680 in Isolated Reaction Centers of Photosystem II.. Chem. Phys.Lett..

[110] Lendzian F, Bittl R, Lubitz W (1998). Pulsed ENDOR of the Photo-Excited Triplet States of Bacteriochlorophyll a and of the Primary Donor P865 in Reaction Centers of Rhodobacter sphaeroides R-26.. Photosynth. Res..

[111] Lendzian F, Bittl R, Telfer A, Lubitz W (2003). Hyperfine Structure of the Photoexcited Triplet State 3P680 in Plant PS II Reaction Centers as Determined by Pulse ENDOR.. Biochim. Biophys. Acta Bioenerg..

[112] Gilbert B, Davies M, Murphy D, Lubitz W (2004). In Electron Paramagnetic Resonance.. A specialist periodical report..

[113] Niklas J, Schulte T, Prakash S, van Gastel M, Hofmann E, Lubitz W (2007). Spin-Density Distribution of the Carotenoid Triplet State in the Peridinin-chlorophyll-protein Antenna.A Q-Band Pulse Electron- Nuclear Double Resonance and Density Functional Theory Study.. J. Am. Chem. Soc..

[114] Rodig C, Siebert F (1999). Error and Artifacts in time-resolved step-scan FT-IR spectroscopy.. Appl. Spectr..

[115] Chandlers JM, Griffiths PR, Rodig C, Siebert F (2002). Instrumental aspects of time-resolved spectra generated using step-scan interferometers in Handbook of Vibrational Spectroscopy.. Wiley.

[116] Rodig C, Georg H, Siebert. F, Rousso I, Sheves M (1999). Temperature effects of excitation laser pulses during step-scan FT-IR experiments.. Laser Chem..

[117] Manning CJ, Griffiths PR (1997). Noise Sources in step-scan FT-IR spectrometry.. Appl Spectr..

[118] Berthomieu C, Hienerwadel R (2009). Fourier transform infrared (FTIR) spectroscopy.. Photosynth Res..

[119] Breton J, Nabedryk E (1993). S T1 infrared difference spectrum of the triplet state of the primary electron donor in Rb.sphaeroides photosynthetic bacterial reaction centers.. Chem. Phys. Lett..

[120] Pinto E, Catalani LH, Peporine Lopes N, Di Mascio P, Colepicolo P (2000). Peridinin as the major biological crotenoid quencher of singlet oxygen in marinae algae Gonyaulax Polyedra.. Biochim. Biophys. Res. Comm..

[121] Breton J, Nabedryk E, Leibl W (1999). FTIR study of the primary electron donor of photosystem I (P700) revealing delocalization of the charge in P700(+) and and localization of the triplet state in 3P700.. Biochemistry.

[122] Sivakumar V, Wang R, Hastings G (2005). A1 reduction in intact cyanobacterial photosystem I particles studied by time-resolved step-scan Fourier transform infrared difference spectroscopy and isotope labeling.. Biochemistry.

[123] Blanchet L, Ruckebusch C, Mezzetti A, Huvenne JP, De Juan A (2009). Monitoring and interpretation of photoinduced biochemical processes by rapid-scan FTIR difference spectroscopy and hybrid hard and soft modeling.. J. Phys. Chem. B.

[124] Mezzetti A, Blanchet L, De Juan A, Leibl W, Ruckebusch C (2011). Ubiquinol formation in isolated photo-synthetic reaction centres monitored by time-resolved differential FTIR in combination with 2D correlation spectroscopy and multivariate curve resolution.. Anal. Bi-oanal. Chem..

[125] Salvadori E, Di Valentin M, Kay CMW, Pedone A, Barone V, Carbonera D (2012). The electronic structure of the lutein triplet state in plant light-harvesting complex II.. Phys. Chem. Chem. Phys..

[126] Fuciman M, Enriquez MM, Kaligotla S, Niedzwiedzki DM, Kajikawa T, Aoki K, Katsumura S, Frank HA (2011). Singlet and Triplet State Spectra and Dynamics of Structurally-modified Peridinins.. J. Phys. Chem. B.

[127] McConnell HM (1961). Intramolecular Charge Transfer in Aromatic Free Radicals.. J. Chem. Phys..

[128] Deligiannakis Y, Louloudi M, Hadjiliadis N (2000). Electron spin echo envelope modulation (ESEEM) spectroscopy as a tool to investigate the coordination environment of metal centers.. Coord. Chem. Rev..

[129] Lin TS (1984). Electron spin echo spectroscopy of organic triplets.. Chem. Rev..

[130] Hsu CP (2009). The electronic couplings in electron transfer and excitation energy transfer.. Acc. Chem. Res..

[131] You ZQ, Hsu CP (2011). Ab Inito Study on Triplet Excitation Energy Transfer in Photosynthetic Light-Harvesting Complexes.. J. Phys. Chem. A..

[132] Mackowski S, Wormke S, Maier A, Brotosudarmo T, Harutyunyan H, Hartschuh A, Govorov A, Scheer H, Brauchle C (2008). Metal-enhanced fluorescence of chlorophylls in single light-harvesting complexes.. Nano Lett..

[133] Czechowski N, Nyga P, Schmidt M, Brotosudarmo T, Scheer H, Piatkowski D, Mackowski S (2012). Absorption enhancement in Peridinin-Chlorophyll-Protein light-harvesting complexes coupled to seemicontinuous Silver Film.. Plasmonics..

[134] Krajnik B, Czechowski N, Ciszak K, Piatkowski D, Mackowski S. Brotosudarmo, T.H.P. Scheer, H. Pichler, S. Heiss W (2012). Plasmon enhanced fluorescence in herterochlorophyllous peridinin-chlorophyll-protein photosyntrhetic. complexes, Opt. Mat..

[135] Kowalska D, Krajnik B, Olejnik M, Twardowska M, Czechowski N, Hofmann E, Mackowsli S (2013). Metal-enhanced fluorescence of chlorophylls in light-harversting complexes coupled to silver nanowires.. Scientific World J..

[136] Krajnik B, Gajda Raczka M, Piatkowski D, Nyga P, Jankiewicz B, Hofmann E, Mackowski S (2013). Silica nanoparticles as a tool for fluorescence collection efficiency enhancement.. Nanoscale Res. Lett..

[137] Mackowski S (2010). Hybrid nanostructures for efficient light harvesting, J.. Phys. Condens. Matter.

[138] Hashim A, Mackowski S (2012). Metallic nanoparticles coupled with photosynthetic complexes in Smart Nanoparticles.. Tech-nology Intechopen..

[139] Andreussi O, Biancardi A, Corni S, Mennucci B (2013). Plasmon-controlled light-harvesting design rules for biohybrid devices via multiscale modeling.. Nano Lett..

[140] Osuka A, Kume T (1998). Fucoxanthin and Peridinin Pheophorbide-a molecules as a new light-harvesting model.. Tetrahedron Lett..

[141] Osuka A, Kume T, Haggquist GW, Javorfi T, Lima JC, Eurico M, Razi Naqvi K (1999). Photophysuical characteristics of two antenna systems a Fucoxanthin Pheophorbide dyad and its peridinin analogue.. Chem Phys Lett..

[142] Polivka T, Pellnor M, Melo E, Pascher T, Sundstrom V, Osuka A, Razi Naqvi K (2007). polarity-tuned Energy transfer efficiency in artificial light-harbvestig antenna containing carbonyl carotenoids peridinin and fucoxanthin.. J. Phys. Chem C..

[143] Recktenwald D (1989). Peridinin-chlorophyll complex as fluorescent label.. U.S. patent 4 876 190 Oct. 24..

[144] Afar B, Merrill J, Clark EA (1991). Detection of Lymphocyte substes using Three-Color/Single Laser flow cytometry and the fluorescent dye Peridinin-Chlorophyll-a-Protein, J.. Clin. Immunol..

[145] Gothot A, Grosdent JC, Paulus JM (1996). A strategy for multiple immunophenotyping by image cytometry model studies using latex microbeads labeled with seven streptavidin-bound fluorochromes.. Cytometry..

[146] Yoshida T, Maoka T, Das SK, Kanazawa K, Horinaka M, Wakada M, Satomi H, Nishiçno H, Sakai T (2007). Halocynthiaxanhin and peridinin sensitize colon cancer cells to tumor necrosisfactor-related apoptosis-inducing ligand.. Mol. Cancer Res..

[147] Sugawara T, Yamashita K, Sakai S, Asai A, Nagazo A, Shiraishi T, Imai I, Hirata T (2007). Induction of apoptosis in DLD-1 human colon cancer cells by peridinin isolated from the diniofalgellate Heterocapsa triquetra.. Biosci. Biotechnol. Biochem..

[148] Wörmke S, Mackowski S, Schaller A, Brotosudarmo TH, Johanning S, Scheer H, Bräuchle C (2008). Single molecule fluorescence of native and refolded peridinin-chlorophyll-protein complexes.. J. Fluoresc..

